# Synthesis and Antibacterial Evaluation of New Pyrazolo[3,4-*d*]pyrimidines Kinase Inhibitors

**DOI:** 10.3390/molecules25225354

**Published:** 2020-11-16

**Authors:** Chiara Greco, Rosa Catania, Dario Leonardo Balacco, Vincenzo Taresco, Francesca Musumeci, Cameron Alexander, Alan Huett, Silvia Schenone

**Affiliations:** 1 Dipartimento di Farmacia, Università di Genova, Viale Benedetto XV 3, 16132 Genova, Italy; greco.phd@difar.unige.it (C.G.); francesca.musumeci@unige.it (F.M.); 2 School of Life Sciences, University of Nottingham, Nottingham NG7 2UH, UK; rosa.catania1@nottingham.ac.uk; 3 School of Dentistry, Institute of Clinical Sciences, College of Medical and Dental Sciences, University of Birmingham, Birmingham B5 7EG, UK; d.l.balacco@bham.ac.uk; 4 School of Chemistry, University of Nottingham, University Park, Nottingham NG7 2RD, UK; vincenzo.taresco@nottingham.ac.uk; 5 School of Pharmacy, University of Nottingham, University Park, Nottingham NG7 2RD, UK; cameron.alexander@nottingham.ac.uk

**Keywords:** pyrazolo[3,4-*d*]pyrimidines, kinase inhibitors, antimicrobials, β-lactam antibiotics

## Abstract

Pyrazolo[3,4-*d*]pyrimidines represent an important class of heterocyclic compounds well-known for their anticancer activity exerted by the inhibition of eukaryotic protein kinases. Recently, pyrazolo[3,4-*d*]pyrimidines have become increasingly attractive for their potential antimicrobial properties. Here, we explored the activity of a library of in-house pyrazolo[3,4-*d*]pyrimidines, targeting human protein kinases, against *Staphylococcus aureus* and *Escherichia coli* and their interaction with ampicillin and kanamycin, representing important classes of clinically used antibiotics. Our results represent a first step towards the potential application of dual active pyrazolo[3,4-*d*]pyrimidine kinase inhibitors in the prevention and treatment of bacterial infections in cancer patients.

## 1. Introduction

Pyrazolo[3,4-*d*]pyrimidine derivatives are a class of bioisosteres of purines that possess a wide variety of pharmacological activities, including antitumor and antimicrobial properties [1].

The combination of anticancer and antibacterial activities in the same molecule can be particularly advantageous during cancer therapy, where vulnerability to bacterial infections increases. However, few studies have examined these dual activities and fewer have sought to optimize for anti-infection effects alongside antitumor efficacy. Cancer-induced immunosuppression; cancer treatments (such as chemotherapy, immunotherapy, radiation therapy or surgery) and other associated procedures (such as long-term vascular catheters) can cause a breakdown in mucosal barriers and increase the susceptibility to bacterial infections [2]. Particularly, it has emerged that *Staphylococcus aureus* has a major clinical impact on patients with malignancy [3].

While the anticancer activity of pyrazolo[3,4-*d*]pyrimidines, exerted by the inhibition of eukaryotic protein kinases, is a well-known and studied therapeutic effect [4], their antimicrobial activity has emerged only recently [5,6,7], and the mechanism of action by which these compounds exert their antibacterial activity has not yet been fully understood.

Since the discovery of protein kinase N1 (PKN1) [8], a bacterial serine/threonine kinase with a high structural homology to eukaryotic protein kinases, sequencing efforts have uncovered the ubiquitous eukaryotic-like serine/threonine kinases (eSTKs) that phosphorylate multiple protein substrates and affect many areas of bacteria cell biology [9]. A subset of eSTKs are single-pass transmembrane proteins that have extracellular penicillin-binding-protein and serine/threonine kinase-associated (PASTA) domains, which bind muropeptides [10]. PASTA domains are small modules that were originally found in one or multiple copies at the C-terminal end of either penicillin-binding proteins (PBPs) or Ser/Thr protein kinases (STPKs) and only later also in different types of proteins [11].

To date, it is known that PASTA kinases are widely distributed in Gram-positive and Gram-negative bacteria [12] and are involved in regulating the resistance to β-lactam antibiotics [13]. Small molecules capable of inhibiting PASTA kinases have been shown to increase pathogen sensitivity to β-lactam antibiotics [14,15]. Besides the efforts in finding prokaryotic kinase inhibitors as possible new antibacterial agents, a large number of pyrazolo[3,4-*d*]pyrimidine derivatives have shown promising activity against bacterial proliferation [16,17,18]. In 2003, Ali A. et al. described the inhibition of *S. aureus* DNA polymerase III and the growth of several other Gram-positive bacteria in culture by pyrazolo[3,4-*d*]pyrimidin-4-one molecules [19]. Later, different compounds characterised by a pyrazolo[3,4-*d*]pyrimidine core have shown antibacterial activity [7]; Khobragade C. et al. reported a set of novel pyrazolo[3,4-*d*]thiazolo[3,2-*a*]pyrimidin-4-one derivatives exhibiting promising antibacterial and antifungal properties on several pathogenic microorganism species [20]. However, a more in-depth investigation into the mechanism of action exerted by this class of compounds on prokaryotic cells is pivotal to improve their antimicrobial activity.

Over recent years, our group has designed and synthesised a wide library of pyrazolo[3,4-*d*]pyrimidines that can target eukaryotic protein kinases and inhibit a variety of cytoplasmic tyrosine or serine-threonine kinases [21,22]. Particularly, our compounds showed in vitro and in vivo anticancer activity with encouraging results for the treatment of a variety of tumours [23,24,25].

Here, we report the antibacterial activity of a representative set of pyrazolo[3,4-*d*]pyrimidines, presenting different substituents in position N1, C4 and C6 against the Gram-positive bacterium *S. aureus* and the Gram-negative bacterium *Escherichia coli*.

## 2. Results and Discussion

**Figure 1:**
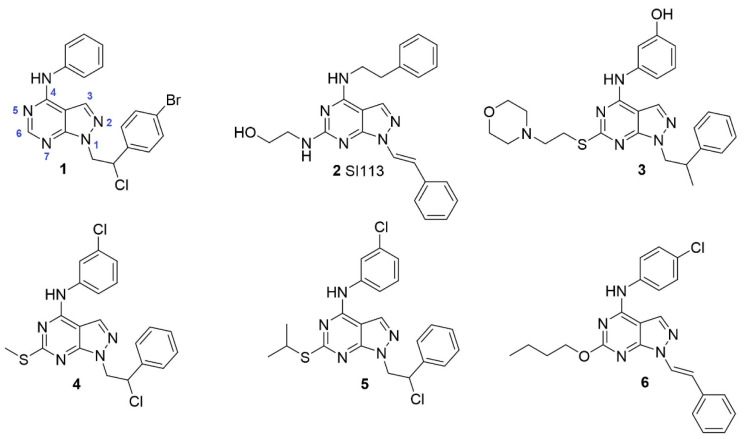
Structure of selected pyrazolo[3,4-*d*]pyrimidines **1**–**6**.

A representative number of pyrazolo[3,4-*d*]pyrimidines, presenting different substituents in positions N1, C4 and C6 and different selectivity against eukaryotic kinases, were evaluated for antibacterial activity. Initially, we selected four pyrazolo[3,4-*d*]pyrimidines (Compounds **1**–**4**, Figure 1) for which we have previously reported significant anticancer properties [21,26,27,28]. Briefly, the C6-unsubstitued pyrazolo[3,4-*d*]pyrimidine **1** demonstrated promising in vivo activity, inducing 50% reductions of tumour volumes in treated mice by blocking Bcr-Abl T315I mutant kinase activity [26]. Compound **2** (called SI113), which acts as an inhibitor of the serine-threonine kinase SGK1, has been widely investigated; several biological studies have further demonstrated its promising activity at low micromolar concentrations, alone or in associative antineoplastic treatments on several types of solid tumours, such as hepatocellular carcinoma [29], glioblastoma multiforme (GBM) [30,31] and ovarian cancer [25]. Interestingly, no toxicity signs were detected in SI113-treated mice [24]. Compounds **3** and **4** are inhibitors of the tyrosine kinase Src showing K_i_ (inhibitory constant) values of 0.07 µM and 0.6 µM, respectively. Compound **3** was evaluated against neuroblastoma cell lines (SH-SY5Y), reporting an IC_50_ value of 0.12 µM, and further demonstrated its low nonspecific cytotoxicity in normal embryonic fibroblasts Wi3827. Compound **4** proved to be active in osteogenic sarcoma, and it was shown to reduce bone resorption in vivo, without interfering with normal osteoblast growth [28]. We then selected two newly synthesised molecules (compound **5** and **6**, Figure 1) characterised by the presence of substituents that could increase the pyrazolo[3,4-*d*]pyrimidine lipophilicity. In fact, compound **5** is a variation of compound **4** in which the thiomethyl group was substituted with the bulkier thioisopropyl group in C6, while compound **6** is a SI113 analogue presenting a buthylether substituent in C6 and a 4-chloroaniline in C4.

Thereafter, we tested this library to evaluate their growth inhibition effect against a pathogenic representative Gram-positive bacterium (*Staphylococcus aureus* Newman) and a Gram-negative bacterium (*Escherichia coli* XL-1). These organisms were selected as representatives of both opportunistic pathogens common in cancer patients (*S. aureus*) [3] and less pathogenic commensal organisms commonly affected by chemotherapy (*E. coli*) [32].

### 2.1. Phylogenetic Analysis of the PASTA Domain-Containing Protein Family

**Figure 2:**
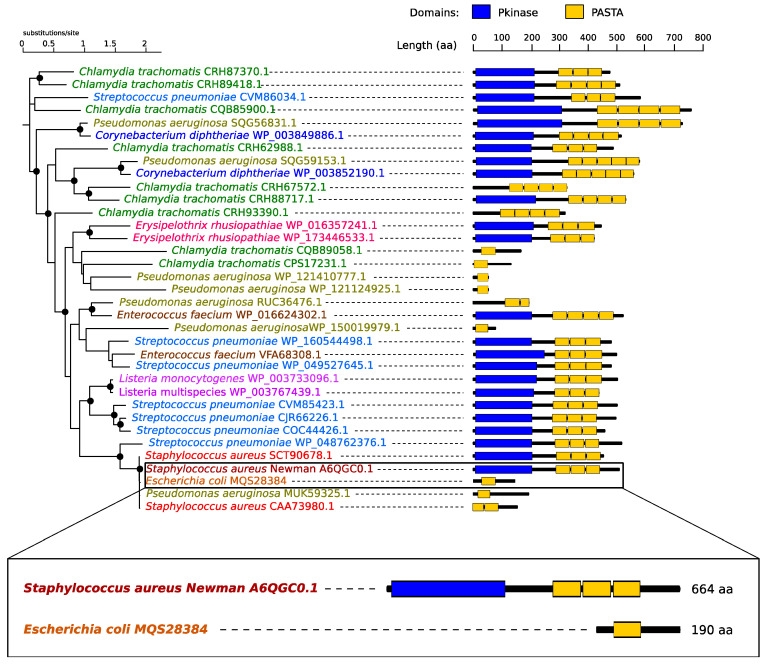
Phylogenetic tree of PASTA domains associated with the relevant bacteria. Predicted protein domains are shown on the right: kinase domains in blue and PASTA domains in yellow. Black circles indicate strongly supported branches (SH-like branch support values > 0.8). Note in the box a schematic of the alignment between the PASTA domain-containing proteins in the species used in this study; *Staphylococcus aureus* Newman has a kinase containing a PASTA domain, whilst *Escherichia coli* lacks the kinase domain.

We reconstructed the phylogenetic tree based on the alignment of eSTK/PASTA domains containing proteins in nine bacterial species (Supplementary Materials Table S1 and Figure S1) demonstrating a wide conservation of the domains (Figure 2). Interestingly, the analysis identified a PASTA domain-containing kinase in *S. aureus* Newman, whilst *E. coli* possessed a short PASTA domain protein and a separate eSTK without a PASTA domain (Figure 2). This suggests that the *S. aureus* protein retained the ability to bind the β-lactam ring of β-lactam antibiotics via its PASTA domain and to phosphorylate Ser/Thr via the eSTK domain. In contrast, the *E. coli* lone PASTA domain can bind to β-lactam antibiotics but is unable to transmit the signal, as it lacks the linked eSTK domain (Figure 2).

It was shown that eSTK-PASTA proteins are involved in cell wall stress signalling and antibiotic responses [13]. We therefore hypothesised that kinase inhibitors would potentiate antibiotics that increased cell wall stress, e.g., ampicillin, in an organism containing an eSTK-PASTA protein. This suggested that *S. aureus* and *E. coli* might exhibit responses to combined drug treatments different from that predicted simply by their single drug responses or cell wall/membrane physiology. We set out to test this idea by exposing bacteria to kinase inhibitors and antibiotics, both singly and in combination, using ampicillin and kanamycin.

### 2.2. Synthesis of Pyrazolo[3,4-d]pyrimidines

**Scheme 1:**
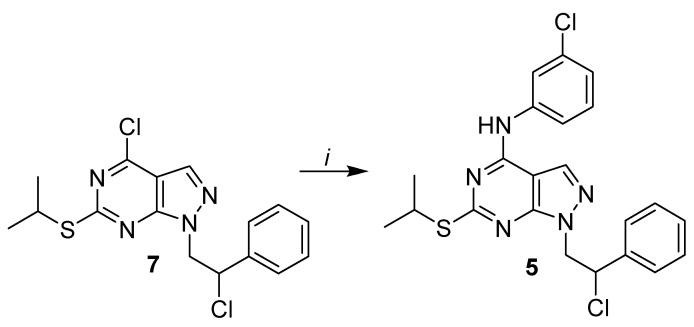
Reagents and conditions: (**i**) 3-chloroaniline, abs. EtOH, reflux, 5 h.

Compounds **1**–**4** were obtained following the synthetic route designed by our research group, as reported previously [21,26,27]. Compound **5**, as shown in Scheme 1, was obtained by performing an aromatic nucleophilic substitution reaction with 3-chloroaniline in absolute ethanol under reflux conditions on the dichloro-C6-thioisopropyl-substituted derivative **7**, previously reported by us [33].

**Scheme 2:**
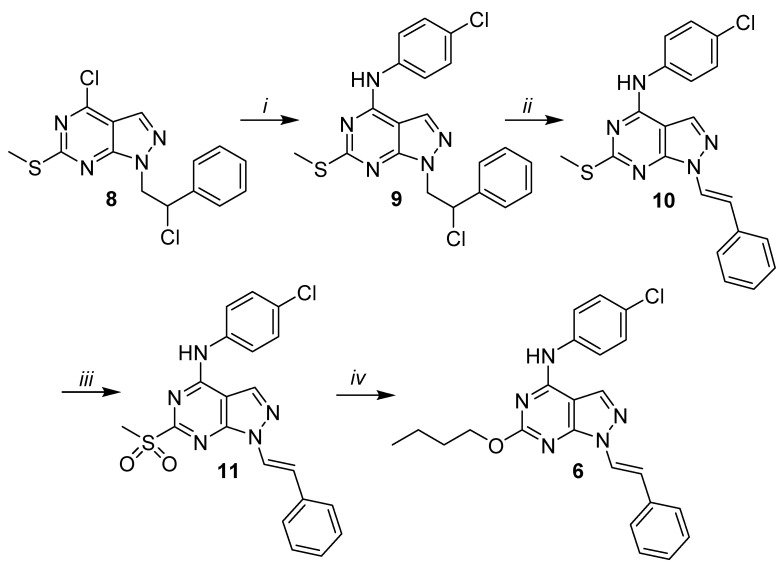
Reagents and conditions: (**i**) 4-chloroaniline, abs. EtOH, reflux, 4 h; (**ii**) 3.3-M aq. NaOH, EtOH 95%, 80 °C, 5 h; (**iii**) mCPBA, an. CHCl3, rt, 6 h and (**iv**) 1-butanol, diethanolamine, dimethyl sulfoxide (DMSO), 100 °C, 24 h.

The synthetic pathway to obtain compound **6** is reported in Scheme 2. Initially, the dichloro-C6-thiomethyl substituted starting derivative **8**, obtained according to our previously published method [34], was reacted with an excess of 4-chloroaniline in absolute ethanol under reflux for 4 h to give compound **9** in good yield. The latter was dehydrohalogenated by refluxing with NaOH for 5 h to obtain N1-styryl derivative **10**. Then, the thiomethyl group of **10** was oxidised by using m-chloroperbenzoic acid in anhydrous CHCl_3_ at room temperature, and subsequently, the sulfone group of derivative **11** was displaced by 1-butanol in the presence of diethanolamine and dimethyl sulfoxide (DMSO) to afford the final compound **6**.

### 2.3. Biological Activities

**Figure 3:**
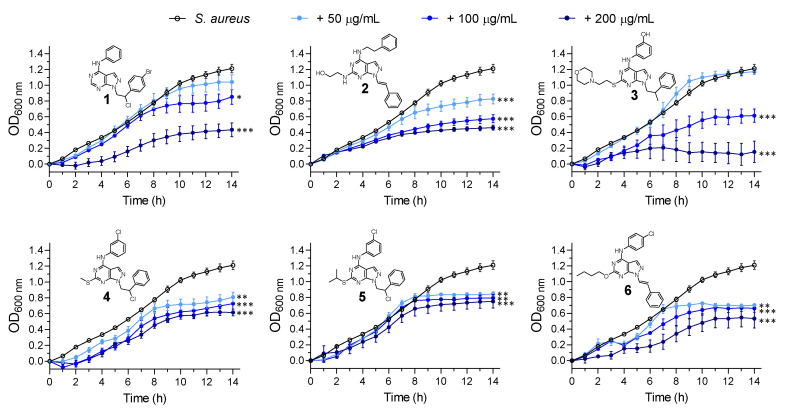
Growth curves of *S. aureus* in the presence of increasing concentrations (50, 100 and 200 µg/mL) of pyrazolo[3,4-*d*]pyrimidines (compounds **1**–**6**). Data are representative of at least 3 independent repeat experiments. Error bars indicate SEM. *p*-values of significance (* < 0.05, ** < 0.01 and *** < 0.001) were determined using one-way analysis of variance (ANOVA). OD: optical density.

**Figure 4:**
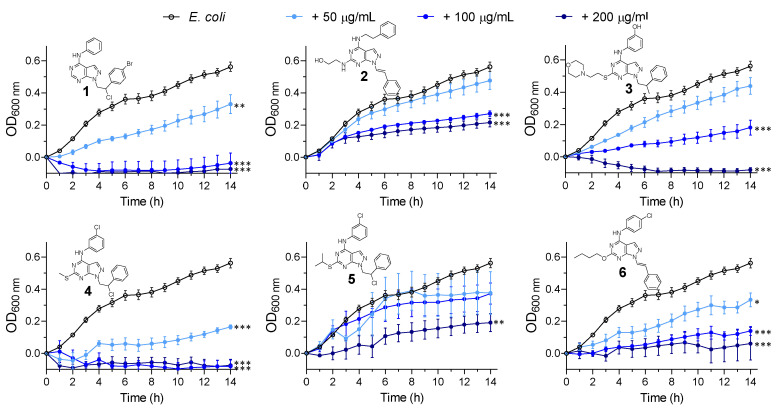
Growth curves of *E. coli* in the presence of increasing concentrations (50, 100 and 200 µg/mL) of pyrazolo[3,4-*d*]pyrimidines (compounds **1**–**6**). *P*-values of significance (* < 0.05, ** < 0.01 and *** < 0.001) were determined using one-way analysis of variance (ANOVA). Data are representative of at least 3 independent repeat experiments. Error bars indicate SEM. OD: optical density.

We next screened our synthesised pyrazolo[3,4-*d*]pyrimidines (**1**–**6**) for their potential antibacterial activity against *S. aureus* and *E. coli*. We exposed the bacteria at increasing concentrations (50, 100 and 200 µg/mL) of pyrazolo[3,4-*d*]pyrimidines (**1**–**6**) and monitored the bacterial growth by measuring the optical density every hour for 14 h. These studies revealed that all the tested compounds displayed significant activities against *S. aureus* (Figure 3) and *E. coli* (Figure 4) in a dose-dependent manner. Compound **3** at 200 µg/mL was capable of almost completely inhibiting *S. aureus* growth (Figure 3). *S. aureus* treated with compounds **4** and **5** showed similar profile growth curves, reflecting the compounds’ structural similarity, characterised by the presence of a meta-substituted aniline in C4.

All compounds showed higher growth inhibition effects against Gram-negative *E. coli*, compared to *S. aureus*. In particular, compound **4** was able to halve *E. coli* bacterial growth (Figure 4) at the lowest concentration tested (50 µg/mL). For compounds **2** (SI113) and **3**, which here showed comparable antimicrobial activity against both the bacterial strains, we have previously reported their anticancer activity in vitro and in vivo [27,35] (as discussed above). Interestingly, the SI113-dependent inhibition of SGK1 was also demonstrated to enhance cytotoxic autophagy in GBM cell lines, leading to cancers cell death [30]. In the prior literature, other antibiotics such as isoniazid or pyrazinamide [36] were reported to activate autophagy, which, in this case, can facilitate host defences against intracellular pathogens and connect the innate and adaptive immune functions [37,38]. It may thus be suggested that an enhancement of autophagic mechanisms mediated by kinase inhibitors could help in the fight against bacterial infections in host cells. Taken together, these results support the potential application of our kinase inhibitors with antitumor/antibacterial dual activity for preventing bacterial infections in oncologic patients [39,40].

### 2.4. Antibiotic Susceptibility Study

**Figure 5:**
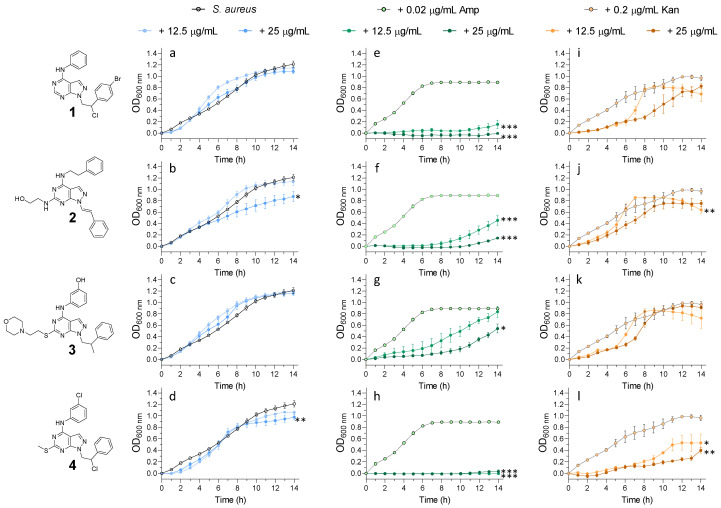
Growth curves of *S. aureus* grown in the presence of (**a**–**d**) pyrazolo[3,4-*d*]pyrimidines (12.5 and 25 μg/mL), (**e**–**h**) pyrazolo[3,4-*d*]pyrimidines (12.5 and 25 μg/mL) in the presence of a sublethal dose of ampicillin (0.02 μg/mL) and (**i**–**l**) pyrazolo[3,4-*d*]pyrimidines (12.5 and 25 μg/mL) in the presence of a sublethal dose of kanamycin (0.2 μg/mL). Amp = ampicillin and Kan = kanamycin. Data are representative of at least 3 independent repeat experiments. Error bars indicate SEM. *p*-values of significance (* < 0.05, ** < 0.01 and *** < 0.001) were determined using one-way analysis of variance (ANOVA). OD: optical density.

We hypothesised that the inhibition of eSTKs by kinase inhibitors should result in a synergistic sensitisation to β-lactam antibiotics against *S. aureus*. To test this hypothesis, we incubated either *S. aureus* and *E. coli* with or without the pyrazolo[3,4-*d*]pyrimidines in the presence of subinhibitory doses of ampicillin and kanamycin. The subinhibitory antibiotic concentrations were determined via broth microdilution assay (Figure S2 from the Supplementary Materials). The concentrations of pyrazolo[3,4-*d*]pyrimidines used in this study (12.5 and 25 μg/mL) had no effect on *S. aureus* growth (Figure 5a–d). Similarly, the subinhibitory concentrations of ampicillin (Figure 5e–h) and kanamycin (Figure 5i–l) had no effect on *S. aureus* growth. However, the treatment of *S. aureus* with subinhibitory concentrations of ampicillin in the presence of 12.5 and 25 μg/mL of pyrazolo[3,4-*d*]pyrimidines led to a significant dose-dependent increase in susceptibility (Figure 5e–h, dark-green lines versus light-green line). Importantly, the susceptibility to kanamycin was unaffected (Figure 5i–l), except from compound **4**, which showed a slight interaction effect. Taken together, these data seem to support our hypothesis that pharmacologic kinase inhibition by pyrazolo[3,4-*d*]pyrimidines in *S. aureus* specifically sensitises bacteria to ampicillin antibiotic.

**Figure 6:**
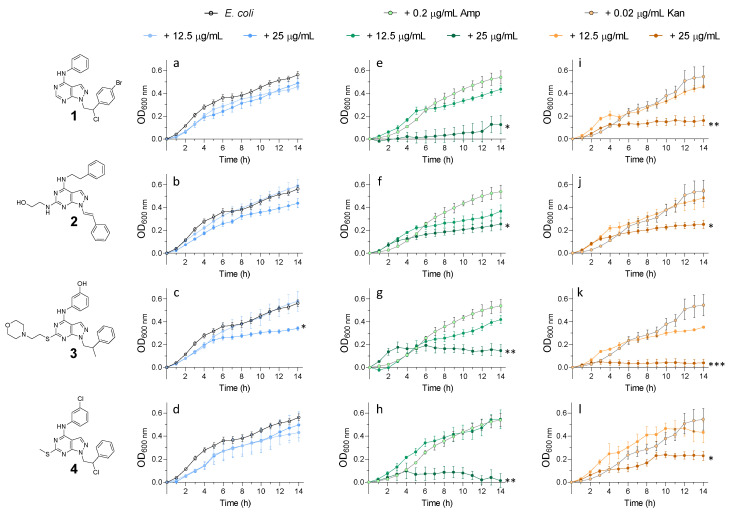
Growth curves of *E. coli* grown in the presence of (**a**–**d**) pyrazolo[3,4-*d*]pyrimidines (12.5 and 25 μg/mL), (**e**–**h**) pyrazolo[3,4-*d*]pyrimidines (12.5 and 25 μg/mL) in the presence of a sublethal dose of ampicillin (0.2 μg/mL) and (**i**–**l**) pyrazolo[3,4-*d*]pyrimidines (12.5 and 25 μg/mL) in the presence of a sublethal dose of kanamycin (0.02 μg/mL). *p*-values of significance (* < 0.05, ** < 0.01 and *** < 0.001) were determined using one-way analysis of variance (ANOVA). Data are representative of at least 3 independent repeat experiments. Error bars indicate SEM. OD: optical density.

On the contrary, when tested against *E. coli* (Figure 6), both susceptibilities to ampicillin and kanamycin were similarly affected only in the presence of 25 μg/mL (highest concentration tested) of pyrazolo[3,4-*d*]pyrimidines (Figure 6).

This data supported our initial hypothesis that, in a bacterium containing kinase-PASTA proteins, kinase inhibition would increase the susceptibility to cell wall stress and, therefore, ampicillin treatment, as seen in *S. aureus* Newman. In *E. coli*, which lacks such a PASTA domain-containing kinase, whilst kinase inhibition did restrict growth, the interaction with ampicillin was not seen for compounds **2** and **3**. Compounds **1** and **4** showed an interaction effect with ampicillin only at the highest concentration tested, similarly to the kanamycin interaction. This suggests that, in *E. coli*, the kinase inhibition affects other cell functions and not the critical cell wall signalling seen in *S. aureus*.

## 3. Materials and Methods

### 3.1. Phylogenetic Analysis of the PASTA Domain-Containing Protein Family

Proteins containing a putative PASTA domain were identified in 9 species (reported in Supplementary Materials Table S1) by protein BLAST (2.2.31+) [41] using the consensus PASTA domain as the query (PFAM accession number PF03793). Sequences in the same species with a similarity higher than 90% were removed. Sequence alignment was performed using MAFFT (v7.266) with the -einsi option [42], and the phylogenetic tree was constructed using FastTreeMP (2.1.9) with the -bionj option [43]. Domains were predicted aligning protein sequences against the reference protein database using phmmer [44].

### 3.2. Chemistry

#### 3.2.1. General Information

Starting materials for the chemical synthesis of pyrazolo[3,4-*d*]pyrimidine derivatives were purchased from Aldrich-Italia (Milan, Italy). Melting points were determined with a Büchi 530 apparatus, uncorrected. ^1^H-NMR spectra were recorded in CDCl_3_ or DMSO-d_6_ by using a Varian Gemini 200 (200 MHz) instrument. Chemical shifts were reported as δ(ppm) relative to tetramethylsilane (TMS) as the internal standard, J in Hz. ^1^H patterns were described using the following abbreviations: s = singlet, d = doublet, t = triplet, quint = quintet, sx = sextet, set = septet, m = multiplet and br s = broad singlet. Chromatographic purifications were performed by columns packed with Merk 60 silica gel, 23–400 mesh, for the flash technique. All target compounds possessed a purity of ≥95%, as verified by elemental analyses by comparison with the theoretical values. Analyses for C, H, N and S were within ±0.4% of the theoretical value.

#### 3.2.2. Synthesis Procedures

Procedure for the synthesis of *N*-(3-chlorophenyl)-1-(2-chloro-2-phenylethyl)-6-(isopropylthio)-1*H*-pyrazolo[3,4-*d*]pyrimidin-4-amine (**5**):

3-Chloroaniline (2.09 mL, 20 mmol) was slowly added to a suspension of 4-chloro-1-(2-chloro-2-phenylethyl)-6-(isopropylthio)-1*H*-pyrazolo[3,4-*d*]pyrimidine **7** (3.67 g, 10 mmol) in absolute EtOH (5 mL), and the mixture was refluxed for 5 h. After cooling, a white solid crystallised; then, it was filtered, washed with water and recrystallised from absolute EtOH.

Yield: 65%; mp: 208–214 °C. ^1^H-NMR (CDCl_3_): δ 1.23 (d, *J* = 6.2 Hz, 3H, CH_3_), 1.25 (d, *J* = 6.2 Hz, 3H, CH_3_), 3.99 (set, *J* = 7.0 Hz, 1H, CHS), 5.00–5.10 (m, 2H, CH_2_N), 6.07–6.13 (m, 1H, CHCl), 7.13–7.52 (m, 8H Ar), 8.08–8.09 (m, 1H Ar) 8.60 (s, 1H, H-3).

Anal. calcd. for C_22_H_21_N_5_Cl_2_S: % C 57.64, H 4.62, N 15.28, S 7.00; found: C 57.64, H 4.88, N 15.28, S 6.79.

Procedure for the synthesis of *N*-(4-chlorophenyl)-1-(2-chloro-2-phenylethyl)-6-(methyltio)-1*H*-pyrazolo[3,4-*d*]pyrimidin-4-amine (**9**):

4-Chloroaniline (0.25 g, 2 mmol) was slowly added to a suspension of 4-chloro-1-(2-chloro-2-phenylethyl)-6-(methylthio)-1*H*-pyrazolo[3,4-*d*]pyrimidine 8 (0.34 g, 1 mmol) in absolute EtOH (6.5 mL), and the mixture was refluxed for 5 h. The EtOH was removed under reduced pressure; then, NaOH 1 M (7 mL) was added, and the solution was washed with water (2 × 15 mL), dried (MgSO_4_), filtered and concentrated under reduced pressure. Compound **9** was crystallised with petroleum ether (bp 40–60 °C)/diethyl ether (1:1), and a solid was obtained. Then, compound **9** was purified by column chromatography (silica gel, 100 mesh) using dichloromethane (DCM)/n-hexane (9:1) as the eluant, to afford the pure product as white solid.

Yield: 75%; mp: 226–228 °C. ^1^H-NMR (CDCl_3_): δ 2.42 (s, 3H, SCH_3_), 4.62–4.84 (m, 2H, CH_2_N), 5.54–5.68 (m, 1H, CHCl), 7.03–8.12 (m, 9H Ar), 8.16 (s, 1H, H-3), 10.16 (s, 1H, NH).

Anal. calcd. for C_20_H_17_Cl_2_N_5_S: % C 55.82, H 3.98, N 16.27, S 7.45 found: C 55.98, H 4.16, N 16.18, S 7.26.

Procedure for the synthesis of *N*-(4-chlorophenyl)-6-(methylthio)-1-[2-phenylvinyl]-1*H*-pyrazolo[3,4-*d*]pyrimidin-4-amine (**10**):

A solution of NaOH (0.28 g, 7.0 mmol) in water (2.1 mL) was added to a suspension of **9** (0.43 g, 1 mmol) in 95% EtOH (10 mL), and the mixture was refluxed for 5 h. After cooling, a white solid crystallised; then, it was filtered and recrystallised from absolute EtOH to give a white solid.

Yields: 71%; mp: 121–123 °C. ^1^H-NMR (DMSO-d_6_): δ 2.60 (s, 3H, SCH_3_), 7.38–7.82 (m, 10H, 9H Ar + =CHAr), 8.00 (d, 1H, *J*_trans_ = 13.8 Hz, NCH=), 8.30 (s, 1H, H-3), 10.32 (br s, 1H, NH).

Anal. calcd. for C_20_H_16_ClN_5_S: % C 60.98, H 4.09, N 17.78, S 8.14; found: C 60.74, H 4.00, N 17.59, S 6.22.

Procedure for the synthesis of *N*-(4-chlorophenyl)-6-(methylsulfonyl)-1-[2-phenylvinyl]-1*H*-pyrazolo[3,4-*d*]pyrimidin-4-amine (**11**):

*m*-Chloroperoxybenzoic acid 77% suspension in mineral oil (0.40 g, 2 mmol) was added portion-wise to a suspension of **10** (0.39 g, 1 mmol) in anhydrous CHCl_3_ (5 mL) at 0 °C. Then, the mixture was stirred at room temperature for 6 h. The solvent was evaporated under reduced pressure, and a solid was formed. The solution was washed with NaHCO_3_ 1 M (30 mL) until it obtained a pH~9, water (2 × 15 mL), dried (MgSO_4_), filtered and concentrated under reduced pressure. Compound **11** was purified by column chromatography (silica gel, 100 mesh) using ethyl acetate/n-hexane (7:3) as the eluant to afford the pure product as a white solid.

Yield: 57%; mp: 225–227 °C. ^1^H-NMR (DMSO-d_6_): δ 3.48 (s, 3H, SO_2_CH_3_), 7.23–7.90 (m, 10H, 9Ar + =CHAr), 7.96 (s, 1H, H-3), 8.10 (d, 1H, *J*_trans_ = 16 Hz, NCH=), 10.89 (s, 1H, NH).

Anal. calcd. for C_20_H_16_ClN_5_O_2_S: %C 56.40, H 3.79, N 16.44, S 7.53; found: C 56.52, H 3.88, N 16.27, S 7.23.

Procedure for the synthesis of *N*-(4-chlorophenyl)-6-butoxy-1-[2-phenylethenyl]-1*H*-pyrazolo[3,4-*d*]pyrimidin-4-amine (**6**):

Diethanolamine (0.2 mL, 2 mmol) was added to a suspension of **11** (0.17 g, 0.4 mmol) in 1-butanol (10 mL) and DMSO (4 mL), and the mixture was heated at 100 °C for 24 h. The solvent was evaporated under reduced pressure. The solution was washed with water (2 × 15 mL), dried (MgSO_4_), filtered and concentrated under reduced pressure. The yellow crude oil was purified by column chromatography (silica gel, 100 mesh) using ethyl acetate/n-hexane (6:4) as the eluant to afford the pure product as a white solid.

Yield: 30%; mp: 196–199 °C. ^1^H-NMR (DMSO-d_6_): δ 0.99 (t, 3H, *J* = 6.3 Hz, CH_3_), 1.48 (sx, 2H, *J* = 6.3 Hz, CH_3_CH_2_), 1.71 (quint, 2H, *J* = 6.3 Hz, CH_2_CH_2_CH_2_), 4.41 (t, 2H, *J* = 6.3 Hz, CH_2_O), 7.28–7.54 (m, 11H, 9Ar + =CHAr + H-3), 7.95 (d, 1H, *J*_trans_ = 16 Hz, NCH=). Anal. calcd. for C_23_H_22_ClN_5_O: % C 65.79, H 5.28, N 16.68; found: C 64.81, H 5.00, N 17.06.

### 3.3. Bacterial Strains and Growth Conditions

Broth microdilution assays were performed according to the guidelines of the Clinical and Laboratory Standard Institute (CLSI), except that our drug stock solutions (20 mg/mL) were prepared in dimethyl sulfoxide (DMSO) instead of in Mueller-Hinton broth (MHB).

*Staphylococcus aureus* Newman is a commonly used strain in animal models of *S. aureus* infection, as it is highly virulent. It lacks antibiotic resistance determinants seen in the majority of current clinical isolates and, thus, represents an opportunity to study the role of our compounds in a susceptible background in this exploratory study. *Escherichia coli* XL-1 is a laboratory strain derived from K-12; it possesses tetracycline resistance due to the presence of the Tn10 transposon on the F plasmid.

Initially, a preinoculum of either *S. aureus* Newman or *E. coli* XL-1 were prepared by picking 2 to 3 colonies from fresh overnight plates into 3 mL of MHB in a 14-mL plastic tube (Falcon^®^) and placed at 37 °C, shaking at 245 rpm for 12–14 h. These cultures of each strain were adjusted appropriately by spectrophotometry at 600 nm to provide 10^5^ colony-forming units (CFU)∙mL^−1^ (as previously determined by viable counting experiments and the creation of strain-specific standard curves) in fresh double-concentrated MHB; 100 μL aliquots of these cell suspensions were mixed with 100 μL of drug solution in phosphate-buffered saline (PBS) to achieve final drug concentrations of 200, 100, 50, 25 or 12.5 μg/mL in a 96-well plate and incubated at 37 °C in an orbital shaker (245 rpm for 14 h). For the antibiotic susceptibility experiments, the procedure was the same, except that the proper concentration of either ampicillin or kanamycin was added to the wells containing 12.5 and 25 μg/mL of each compound.

The growth kinetics of each microorganism was determined by measuring the absorbance at 600 nm in an Epoch 2 incubated, shaking microplate reader (BioTek, v2.06.10). The controls were (1) culture media, (2) culture media plus bacteria, (3) culture media plus bacteria with 2% of DMSO, (equivalent to the amount of DMSO in the 200 μg/mL concentration of drugs), (4) culture media plus bacteria and relevant antibiotics and (5) culture media plus bacteria and gentamicin. Values given were averages from at least three independent repeats ± standard error. Statistical comparisons were determined by one-way analysis of variance (ANOVA) using GraphPad Prism (version 8.0). In the figures, statistical probability was indicated by * *p* < 0.05, ** *p* <0.01 and *** *p* <0.001.

## 4. Conclusions

Our preliminary study in an antimicrobial context revealed the strong antibacterial potential of pyrazolo[3,4-*d*]pyrimidines already active as kinase inhibitors [35,45] and endowed with antiproliferative properties. In particular, six compounds were identified for their bacteriostatic activity against Gram-positive *S. aureus* and Gram-negative *E. coli.* We highlighted a favourable combination with ampicillin, suggesting a mechanism whereby increasing the cell wall stress via kinase inhibition increased the susceptibility to cell wall-targeting agents. This interaction was particularly striking in *S. aureus*, which possessed a complete PASTA kinase capable of binding beta-lactams (or other cell wall intermediates) and signalling to the cellular machinery to trigger cell wall repair or modification. This fitted our initial model, whereby inhibition of the PASTA kinase would enhance the susceptibility to cell wall-active agents by blunting the cellular response to cell wall damage.

We originally selected kanamycin as a second antibiotic, as, unlike beta-lactams, it does not directly target the cell wall, rather acting via ribosomal inhibition, the induction of mistranslation and membrane alterations [46]. We anticipated it, therefore, having little interaction with kinase-inhibitory compounds, which would be anticipated to affect mainly peptidoglycan. However, our data indicated that, in both *S. aureus* and *E. coli*, it can nevertheless interact with our kinase inhibitors. This combined effect of kanamycin was less marked that than of ampicillin in *S. aureus*, but the two antibiotics behaved more similarly in *E. coli*. There are several possible explanations for this effect, including that the combined stresses of ribosome dysfunction and eSTK kinase inhibition results in enhancing their effects in a nonspecific manner. Indeed, one could speculate that there may be many pathways involved in responding to kinase inhibition-induced cell wall stress that would require protein synthesis, and diminishing these responses would result in poorer outcomes for the bacteria affected. Another possibility is that kinase inhibitors induce cell wall defects that result in increased membrane permeability, and this allows greater access for kanamycin and, thus, a more effective delivery of antibiotic to its target. Such membrane permeability-based positive feedback was proposed for aminoglycosides, and recent data shows that mistranslated proteins accumulating in membranes may cause leakage and membrane hyperpolarisation [47]. These possible mechanisms and potential synergies caused by interactions between the cell wall and membrane integrity deserve further study in a variety of cells, including defined PASTA kinase knockouts or mutants.

There is currently great interest in the role of the microbiota during cancer therapy, particularly drug side effects (nausea, weight loss and gastrointestinal disturbance), as well as overall recovery. Some evidence suggests that maintaining or reconstituting the intestinal microbiota can improve patient tolerance to oncological therapy and outcomes [48,49]. Therefore, our promising results need to be further explored; the dual activity of pyrazolo[3,4-*d*]pyrimidines could be useful to fight infections in oncologic patients. However, it needs to be remembered that agents capable of targeting bacterial eSTKs also could result in increased, unintended microbiome disruption. The selectivity shown in this initial study may be further improved to offer targeted therapies that avoid major microbiome alterations, preserving gastrointestinal microbiome function and diversity during therapy. There may also be scope to evolve these compounds in different directions, tailoring their activities to offer either powerful antibacterial action or microbiome-sparing anticancer selectivity.

## Supplementary Materials

The following are available online: Table S1: Species name and their taxonomic identifier for the 10 organisms analysed. Figure S1: Alignment of PASTA domain-containing proteins. Figure S2: Antibiotic doses screening for the selection of subinhibitory doses of ampicillin and kanamycin against *S. aureus* and *E. coli.*
