# Supplementary material for: Synthesis and Antibacterial Evaluation of New Pyrazolo[3,4-d]pyrimidines Kinase Inhibitors
Source: Molecules. 2020 Nov 16;25(22):5354. doi: 10.3390/molecules25225354 (PMC7696985; doi:10.3390/molecules25225354)
Supplement: Supplementary file 1 [file molecules-25-05354-s001.zip › Figure S1.pdf]

```
                                                1
StAu_newman_A6QGC0.1                            ----MIGKII NERYKIVDKL GGGGMSTVYL AEDTILN-IK
Stau_aureus_CAA73980.1                          ---------- ---------- ---------- ----------
Ecoli_MQS28384                                  ---------- ---------- ---------- ----------
PsAe_MUK59325.1                                 ---------- ---------- ---------- ----------
Stau_SCT90678.1                                 ----MIGKII NERYKIVDKL GGGGMSTVYL AEDTILN-IK
Stpn_Bacilli_multispecies_WP_048762376.1        ----MNGKLI NDRYQLLDKL GGGGMSIVYL AEDTILE-RK
PsAe_WP_150019979.1                             ---------- ---------- ---------- ------DEPE
PsAe_WP_121410777.1                             ---------- ---------- ---------- ----------
Enfu_VFA68308.1                                 --MIQIGKLF AGRYRILKSI GRGGMADVYL ARDLILDNEE
Stpn_WP_160544498.1                             --MIQIGKLF AGRYRILKSI GRGGMDDVYL AHDLILDNEE
Stpn_Streptococcus_multispecies_WP_049527645.1  --MIQVGKLF AGRYRILKAI GRGGMADVYL ANDLILDNEK
PsAe_RUC36476.1                                 ---------- ---------- ---------- ----------
Enfu_WP_016624302.1                             --MIELGKKL NGRYQITGNI GSGGMANVYL AHDLILD-RD
Limo_WP_003733096.1                             ---MMIGKRL SDRYKILHAI GGGGMANVYL AHDIILD-RD
Listeria_multispecies_WP_003767439.1            ---MMIGKRL NDRYKILHAI GGGGMANVYL AHDIILD-RD
Stpn_CJR66226.1                                 ---MLIGKRI SGRYQILRVI GGGGMANVYL AEDIILD-RE
Stpn_COC44426.1                                 ---MLTGKRI SGRYHILRPI GGGGMANVFL AEDIILE-RE
Stpn_CVM85423.1                                 ---MLIGKRI SGRYQILRAI GGGGMANVYL AEDMILD-RE
Stpn_CVM86034.1                                 -MSVDEQRTL NGRYRIGELI GRGGMADVFR GEDLRLH-RP
Chtr_CQB85900.1                                 MSNTMPTSLA NGRYQLGELI GRGGMAEVRI AEDKRLG-RT
Chtr_CRH87370.1                                 --MTDIPHIL GGRYEVGDLI GRGGMAQVHI GYDTRLS-RT
Chtr_CRH89418.1                                 -MADSMGRRL AGRYEVRSLI GRGGMAEVHL GFDTRLS-RV
PsAe_WP_121124925.1                             ---------- ---------- ---------- ----------
Errh_WP_016357241.1                             -----MNSII GERYRIVKKI GTGGMADVYL ALDTVLN-RE
Errh_WP_173446533.1                             --MPLAVQEV ANRYEIISLI GQGGMADVYK AKDIILN-RI
Chtr_CRH67572.1                                 ---------- ---------- ---------- ----------
Chtr_CRH88717.1                                 MLDPLLATVI DGRYRIEARL ARGGMASVYR GIDTRLQ-RP
PsAe_SQG56831.1                                 -----MNDVI AGRYELGEVI GTGGMSEVYS ATDTLLG-RE
Codi_WP_003849886.1                             ----MTDIVL ADRYRLGDVI GTGGMSEVYE ATDVLLG-RK
Chtr_CRH62988.1                                 ----MPDKVL GGRYIIQDKI GTGGMAIVYR GIDNRLG-RP
PsAe_SQG59153.1                                 MAELIVGDVL ENRYRIEAPI ARGGMSTVYR CLDLRLE-RH
Codi_WP_003852190.1                             MADLKIGDVL ENRYRIEAPI ARGGMSTVYR CVDTRLG-RL
Chtr_CRH93390.1                                 ---------- ---------- ---------- ----------
Chtr_CPS17231.1                                 ---------- ---------- ---------- ----------
Chtr_CQB89058.1                                 ---------- ---------- ---------- ----------

                                               41
StAu_newman_A6QGC0.1                            VAIKAIFIPP REKEETLKRF EREVHNSSQL SHQNIVSMID
Stau_aureus_CAA73980.1                          ---------- ---------- ---------- ----------
Ecoli_MQS28384                                  ---------- ---------- ---------- ----------
PsAe_MUK59325.1                                 ---------- ---------- ---------- ----------
Stau_SCT90678.1                                 VAIKAIFIPP REKEETLKRF EREVHNSSQL SHQNIVSMID
Stpn_Bacilli_multispecies_WP_048762376.1        VAIKAISISQ NEKDETMKRF YREVNSATQI VHENIVEVYD
PsAe_WP_150019979.1                             VAV------- ---------- ---------- ----------
PsAe_WP_121410777.1                             ---------- ---------- ---------- ----------
Enfu_VFA68308.1                                 VAIKVLRTNY QTDQIAVARF QREARAMAEL THPNIVAIRD
Stpn_WP_160544498.1                             VAIKVLRTNY QTDQIAVARF QREARAMAEL NHPNIVAIRD
Stpn_Streptococcus_multispecies_WP_049527645.1  VAIKVLRTNY QTDQVAVARF QREARAMAEL SHPNIVAIRD
PsAe_RUC36476.1                                 ---------- ---------- ---------- ----------
Enfu_WP_016624302.1                             VAIKVLRFDF QNDQTAIRRF QREALAATEL VHPNIVSVYD
Limo_WP_003733096.1                             VAVKILRIDL ADESNLIRRF QREAQSATSL VHPNIVSVYD
Listeria_multispecies_WP_003767439.1            VAVKILRIDL ADESNLIRRF QREAQSATSL VHPNIVSVYD
Stpn_CJR66226.1                                 VAIKILRFDY ANDNEFIRRF RREAQSASSL DHPNIVSIYD
Stpn_COC44426.1                                 VAIKILRFDF VNDIDFIRRF RREAQSASSL DHPNIVSIYD
Stpn_CVM85423.1                                 VAIKILRFDF ASDDDFIRRF RREAQSAASL DHPNIVSIYD
Stpn_CVM86034.1                                 VAVKLMRRDL ARDPHFQARF RKEARSAASL NHPSIVSVYD
Chtr_CQB85900.1                                 VAVKIMRSDL ANDEIFLSRF RREAHSIAQM NNPNIVNIYD
Chtr_CRH87370.1                                 IAIKILRSDL SSDPTFLARF RREAQSAAAL NHPAIVAVYD
Chtr_CRH89418.1                                 VAIKMLRSDL ARDSVFQARF RREAQSAASL NHANIVAVYD
PsAe_WP_121124925.1                             ---------- ---------- ---------- ----------
Errh_WP_016357241.1                             VALKVLRGDL SHDPVALLRF QREANAASGL NHQSIVEVYD
Errh_WP_173446533.1                             VAIKILREKL SHDAMTLVRF QREASAASRL SHPNVVDIYD
Chtr_CRH67572.1                                 ---------- ---------- ---------- ----------
Chtr_CRH88717.1                                 VALKLIHSHL AEQPDFAQRF IREAQAAAAL SNPHIVSIYD
PsAe_SQG56831.1                                 VAIKMLRADL ARDVNFRERF RREAQNSGKL NHPAIVAVYD
Codi_WP_003849886.1                             VAVKMLRADL ARDVNFRERF RREAQNSGKL NHPAIVAVYD
Chtr_CRH62988.1                                 IAIKTMLPQY AADPSFAARF KQEAQAAAAL QSPFIVSVYD
PsAe_SQG59153.1                                 VAAKVMDARY IDDPIFRQRF QREARSMAQL SHPCLVGVYD
Codi_WP_003852190.1                             VAAKVMDQRY LDDPIFKDRF RREARSMAQL SHPCLVGVYD
Chtr_CRH93390.1                                 ---------- ---------- ---------- ----------
Chtr_CPS17231.1                                 ---------- ---------- ---------- ----------
Chtr_CQB89058.1                                 ---------- ---------- ---------M RRPTL-----
```

```
                                                81
StAu_newman_A6QGC0.1                            VD----EED- --------DC YYLVMEYIEG PTLSEYIESH
Stau_aureus_CAA73980.1                          ---------- ---------- ---------- ----------
Ecoli_MQS28384                                  ---------- ---------- ---------- ----------
PsAe_MUK59325.1                                 ---------- ---------- ---------- ----------
Stau_SCT90678.1                                 VD----EED- --------DC YYLVMEYIEG PTLSEYIQSH
Stpn_Bacilli_multispecies_WP_048762376.1        VE----EDD- --------DN FYLIMEYIDG PTLSEYIHSH
PsAe_WP_150019979.1                             ---------- ---------- ---------- ----------
PsAe_WP_121410777.1                             ---------- ---------- ---------- ----------
Enfu_VFA68308.1                                 IG----EED- --------GQ QFLVMEYVDG FDLKKYIQDN
Stpn_WP_160544498.1                             IG----EED- --------GQ QFLAMEYVNG PDLKKYIQDH
Stpn_Streptococcus_multispecies_WP_049527645.1  IG----EED- --------GQ QFLVMEYVDG SDLKKYIQDH
PsAe_RUC36476.1                                 ---------- ---------- ---------- ----------
Enfu_WP_016624302.1                             VG----EES- --------GM QYLVMEYVKG MDLKRYIQTH
Limo_WP_003733096.1                             VG----EEN- --------DL HYIVMEHVDG MDLKQYIHEN
Listeria_multispecies_WP_003767439.1            VG----EEN- --------DL HYIVMEHVDG MDLKQYIQEN
Stpn_CJR66226.1                                 LG----EED- --------DI YYIVMEYVEG MTLKEYITAN
Stpn_COC44426.1                                 IG----EEG- --------DI YYIVMEYVEG MTLKEYITAH
Stpn_CVM85423.1                                 VG----EED- --------DI YYIVMEYVEG MTLKEYIHAN
Stpn_CVM86034.1                                 TG----EVSL ED-GLHPVDC PFLVMELVSG RTLREILHAE
Chtr_CQB85900.1                                 SG----EESV EDDNGVEEHL PYLVMEYVKG KTLRDILKMN
Chtr_CRH87370.1                                 TG----EETI VSDSGRTLAL PYIVMEYVKG RTVSTLLKHG
Chtr_CRH89418.1                                 TG----EEML TGPEGRAISV PYIVMEYVEG HTVKELLADG
PsAe_WP_121124925.1                             ---------- ---------- ---------- ----------
Errh_WP_016357241.1                             VG----EDE- --------GQ HYIVMEMIRG TTLKQLVHRR
Errh_WP_173446533.1                             VG----ESD- --------GM HYIVMEFIRG RTLKQLIQQR
Chtr_CRH67572.1                                 ---------- ---------- ---------- ----------
Chtr_CRH88717.1                                 QGIYHSSEG- --------ER AYLVMELING PDLRSELSAH
PsAe_SQG56831.1                                 TG----ETER AG-----IST PYIVMERVHG RTLRDIVRED
Codi_WP_003849886.1                             TG----ETPR AG-----LNT PYIVMELVNG RTLRDIVRED
Chtr_CRH62988.1                                 WG----KDN- --------DT YFIAMEYLRG TDLKSGVRKH
PsAe_SQG59153.1                                 FS----SDG- --------DQ IFLIMELITG GTLRELLAER
Codi_WP_003852190.1                             FS----SDG- --------DN IFLIMELITG GTLRELLAER
Chtr_CRH93390.1                                 ---------- ---------- ---------- ----------
Chtr_CPS17231.1                                 ---------- ---------- ---------- ----------
Chtr_CQB89058.1                                 ---------- ---------- ---------- ----------

                                               121
StAu_newman_A6QGC0.1                            GPLSVDTAIN FTNQILDGIK HAHDMRIVHR DIKPQNILID
Stau_aureus_CAA73980.1                          ---------- ---------- ---------- ----------
Ecoli_MQS28384                                  ---------- ---------- ---------- ----------
PsAe_MUK59325.1                                 ---------- ---------- ---------- ----------
Stau_SCT90678.1                                 GPLSVDTAIN FTNQILDGIK HAHDMRIVHR DIKPQNILID
Stpn_Bacilli_multispecies_WP_048762376.1        GPLNIETAID FIKQILNGVK QAHEQRIIHR DIKPQNVLIN
PsAe_WP_150019979.1                             ---------- ---------- ---------- ----------
PsAe_WP_121410777.1                             ---------- ---------- ---------- ----------
Enfu_VFA68308.1                                 APLSNNEVVR IMNEVLSAMS LAHQKGIVHR DLKPQNILLT
Stpn_WP_160544498.1                             APLSNTETVR IMSEVLSAMQ LAHQKGIVHR DLKPQNVLLT
Stpn_Streptococcus_multispecies_WP_049527645.1  APLSNQDVVR IMGEILSAMT LAHQKGIIHR DLKPQNVLLT
PsAe_RUC36476.1                                 ---------- ---------- ---------- ----------
Enfu_WP_016624302.1                             YPIPYQTVVD IMQQILSAIS LAHAHRIIHR DLKPQNILID
Limo_WP_003733096.1                             HPISYEKAVD IMLQIVSAVA IAHQHHIIHR DLKPQNILID
Listeria_multispecies_WP_003767439.1            HPISYEKAVD IMLQIVSAVA IAHQHHIIHR DLKPQNILID
Stpn_CJR66226.1                                 GPLHPKEALN IMEQIVSAIA HAHQNQIVHR DIKPHNILID
Stpn_COC44426.1                                 GPLHPKEALS VMEQIVSAIA HAHQNHIVHR DIKPHNILID
Stpn_CVM85423.1                                 GPLHPKEAVR IMEQVVAAME EAHAKQLVHR DIKPHNILID
Stpn_CVM86034.1                                 GAVGTDRAVA WTRGVLEALE HAHEEGIVHR DVKPANVMVT
Chtr_CQB85900.1                                 GALSQRDSEQ VMLGVLNALE YSHRMGVIHR DIKPGNIMIS
Chtr_CRH87370.1                                 QALPINEAVQ IAVGVLSALE YSHYEGIIHR DIKPANIMIS
Chtr_CRH89418.1                                 TPVPINEAVA IVSGVLGALE YSHSQHLVHR DIKPGNIMLT
PsAe_WP_121124925.1                             ---------- ---------- ---------- ----------
Errh_WP_016357241.1                             GALDKYESVA IMQQLASALQ HAHAAHVIHR DIKPQNILVK
Errh_WP_173446533.1                             GALSVSESIQ IMTQLTSAID HAHKHNIIHR DIKPQNVLVK
Chtr_CRH67572.1                                 ---------- ---------- ---------- ----------
Chtr_CRH88717.1                                 GSFTLRDSLE ITRQVLTALA VAHEAGIVHR DVKPENILLS
PsAe_SQG56831.1                                 GPLSPTEAAQ TLLPVCQALQ FSHDAGIIHR DIKPANVMIT
Codi_WP_003849886.1                             GPLTPSQAAH TLIPVCHALQ VSHDAGIIHR DIKPANVMIT
Chtr_CRH62988.1                                 GALDCKKVAQ IGSQIAQALS VAHRHDIIHR DIKPQNIMVQ
PsAe_SQG59153.1                                 GPMPPHAAAA VMHSVLTGLS VAHNAGMVHR DIKPDNVLIN
Codi_WP_003852190.1                             GPMPPHAAVA VMRSVLTGLS VAHSAGMVHR DIKPDNVLIN
Chtr_CRH93390.1                                 ---------- ---------- ---------- ----------
Chtr_CPS17231.1                                 ---------- ---------- ---------- ----------
Chtr_CQB89058.1                                 ---------- ---------- ------LIH- ----------
```

```
                                             161
StAu_newman_A6QGC0.1                         S--------- --NKTLKIFD FGIAKAL--S ETSLTQTNHV
Stau_aureus_CAA73980.1                       ---------- ---------- ---------- ----------
Ecoli_MQS28384                               ---------- ---------- ---------- ----------
PsAe_MUK59325.1                              ---------- ---------- ---------- ----------
Stau_SCT90678.1                              S--------- --NKTLKIFD FGIAKAL--S ETSLTQTNHV
Stpn_Bacilli_multispecies_WP_048762376.1     K--------- --DKILKIFD FGIAKAL--S ETSMTQTNHV
PsAe_WP_150019979.1                          ---------- ---------D FGSVVAA--P YVQM------
PsAe_WP_121410777.1                          ---------- ---------- ---------- ------TNTL
Enfu_VFA68308.1                              K--------- --KGTVKVTD FGIAVAF--A ETSLTQTNSM
Stpn_WP_160544498.1                          K--------- --DGVAKVTD FGIAVAF--A ETSLTQTNSM
Stpn_Streptococcus_multispecies_WP_049527645.1 K--------- --DGRAKVTD FGIAVAF--A ETSLTQTNSM
PsAe_RUC36476.1                              ---------- ---------- ---------- ----------
Enfu_WP_016624302.1                          Q--------- --EGVVKITD FGIAIAL--S ETSITQTNSM
Limo_WP_003733096.1                          H--------- --DGVVKITD FGIAMAL--S ETSITQTNSL
Listeria_multispecies_WP_003767439.1         H--------- --DGVVKITD FGIAMAL--S ETSITQTNSL
Stpn_CJR66226.1                              H--------- --MGNIKVTD FGIATAL--S STTITHTNSV
Stpn_COC44426.1                              H--------- --LGHIKVTD FGIATAL--T STTITHTNSV
Stpn_CVM85423.1                              N--------- --LGNIKVTD FGIAMAL--S SATITHTNSV
Stpn_CVM86034.1                              E--------- --TGAVKVMD FGIAHALADT SATTSQTQAV
Chtr_CQB85900.1                              E--------- --QGIVKVMD FGIARALDDS ATTMTQSQGV
Chtr_CRH87370.1                              Q--------- --DGKVKVMD FGIARAIADS SATMTSTNSV
Chtr_CRH89418.1                              N--------- --DGKVKVMD FGIARAITDS QATMTQTNAV
PsAe_WP_121124925.1                          ---------- ---------- ---------- ----------
Errh_WP_016357241.1                          D--------- --DGTVKITD FGIALAG--D AIQLTKSDSV
Errh_WP_173446533.1                          D--------- --DGTVKITD FGIAIAN--D AVQLTLNNAV
Chtr_CRH67572.1                              ---------- ---------- ---------- ----------
Chtr_CRH88717.1                              ESISHKHVLS PPSYTAKVAD FGLARAV--S DVTSTHSGQM
PsAe_SQG56831.1                              N--------- --TGTVKIMD FGIARALDDA TSAMTQTSAV
Codi_WP_003849886.1                          N--------- --TGAVKIMD FGIARALDDA TSAMTQTSAV
Chtr_CRH62988.1                              P--------- --DGNIKVMD FGIARA---K NSHLTTDNSV
PsAe_SQG59153.1                              S--------- --DHQVKLAD FGLVRAA--S ASQAT-SANI
Codi_WP_003852190.1                          A--------- --DHQVKLAD FGLVRAA--S ASQAT-SNQI
Chtr_CRH93390.1                              ---------- ---------- ---------- ----------
Chtr_CPS17231.1                              ---------- ---------- ---------- ----------
Chtr_CQB89058.1                              ---------- ---------- ---------- ----------

                                             201
StAu_newman_A6QGC0.1                         LGTVQYFSPE QAKGEATDEC TDIYSIGIVL YEMLVGEPPF
Stau_aureus_CAA73980.1                       ---------- ---------- ---------- ----------
Ecoli_MQS28384                               ---------- ---------- ---------- ----------
PsAe_MUK59325.1                              ---------- ---------- ---------- ----------
Stau_SCT90678.1                              LGTVQYFSPE QAKGEATDEC TDIYSIGIVL YEMLVGEPPF
Stpn_Bacilli_multispecies_WP_048762376.1     LGTVQYLSPE QAKGDKTNET TDIYSIGIVL YEMLVGEPPF
PsAe_WP_150019979.1                          ---------- ---------- ---------- --ILKDTLPY
PsAe_WP_121410777.1                          --------PE PKPG------ ---------- ----------
Enfu_VFA68308.1                              LGSVHYLSPE QARGSKATVQ SDIYAMGIML FEMLTGHIPY
Stpn_WP_160544498.1                          LGSVHYLSPE QARGSKATIQ SDIYAMGIML FEMLTCHIPF
Stpn_Streptococcus_multispecies_WP_049527645.1 LGSVHYLSPE QARGSKATIQ SDIYAMGIML FEMLTGRIPY
PsAe_RUC36476.1                              ---------- ---------- ---------- ----------
Enfu_WP_016624302.1                          LGSVHYLSPE QARGSMATKQ SDIYALGIIL YEMLTGSVPF
Limo_WP_003733096.1                          LGSVHYLSPE QARGGMATQK SDIYSLGIVL YELLTGKVPF
Listeria_multispecies_WP_003767439.1         LGSVHYLSPE QARGGMATQK SDIYSLGIVL YELLTGKVPF
Stpn_CJR66226.1                              LGSVHYLSPE QARGGLATKK SDIYALGIVL FELLTGRIPF
Stpn_COC44426.1                              LGSVHYLSPE QARGGLATKK SDIYALGIVL FELLTGKIPF
Stpn_CVM85423.1                              LGSVHYLSPE QARGGLATKK SDIYSLGIVL YELISGRMPF
Stpn_CVM86034.1                              VGTALYLAPE QATGRTVDGR ADLYAAGCLL FELLTGRPPF
Chtr_CQB85900.1                              VGTAQYLSPE QARGEQVDMR SDLYSAGCVL YEMLTGRPPF
Chtr_CRH87370.1                              VGTAQYLSPE QARGEVVDAR SDLYSTGCLI YELLTGKPPF
Chtr_CRH89418.1                              VGTAQYLSPE QARGEQVDAR SDLYSTGVVL FELLTGRPPF
PsAe_WP_121124925.1                          ---------- ---------- ---------- ----------
Errh_WP_016357241.1                          LGSVHYMAPE CSRGEGAGEQ SDVYSLGVVF YELLTGDVPY
Errh_WP_173446533.1                          MGSAHYLAPE TAQGKEPTAQ VDIYSLGIVF YELLTGDVPF
Chtr_CRH67572.1                              ---------- ---------- ---------- -------MPW
Chtr_CRH88717.1                              LGTVAYTAPE IVTRGRADQR ADLYALGVML YELLAGTQPF
PsAe_SQG56831.1                              IGTAQYLSPE QARGKLADAR SDVYALGCVL YETLTGRPPF
Codi_WP_003849886.1                          IGTAQYLSPE QARGKLADAR SDVYALGCVL YETLTGKPPF
Chtr_CRH62988.1                              LGTAHYVSPE QTQGKPLGPT TDIYSLGIVM YEAATGVVPF
PsAe_SQG59153.1                              IGTVSYLSPE QVSGDDIGPA SDVYSAGILL YELLTGTTPF
Codi_WP_003852190.1                          VGTVSYLSPE QVSGDDIGPE SDVYSAGIVM FELLTGTTPF
Chtr_CRH93390.1                              ---------- ---------- ---------- ----------
Chtr_CPS17231.1                              ---------- ---------- ---------- ----------
Chtr_CQB89058.1                              ---------- ---------- -----IGLM- ----------
```

```
                                                  241
StAu_newman_A6QGC0.1                               NGETAVSIAI KHIQDSVPNV T---TDVRKD IPQSLSNVIL
Stau_aureus_CAA73980.1                             ---------- ---------- ---------- ----------
Ecoli_MQS28384                                     ---------- ---------- ---------- ----------
PsAe_MUK59325.1                                    ---------- ---------- ---------- ----------
Stau_SCT90678.1                                    NGETAVSIAI KHIQDSVPNV T---TDVRKD IPQSLSNVIL
Stpn_Bacilli_multispecies_WP_048762376.1           RGETAVSIAI KHIQETVPNI ----TEKHPN IPQSLSNVVL
PsAe_WP_150019979.1                                LG-------- ---------- ---------- ----------
PsAe_WP_121410777.1                                ---------- ---------- ---------- ----------
Enfu_VFA68308.1                                    DGDSAVTIAL QHFQKPLPSI ----LAENKS VPQALENIVI
Stpn_WP_160544498.1                                DGDSAVTIAL QHFQKPLPSI ----IFENRN VPQALENVVI
Stpn_Streptococcus_multispecies_WP_049527645.1     DGDSAVTIAL QHFQKPLPSI ----LAENHN VPQALENVVI
PsAe_RUC36476.1                                    ----AVTIAL KHFQEEIPSV ----KMFDPG IPQSLENVVR
Enfu_WP_016624302.1                                DGESAVTIAL KHFQEDLPSI ----TTLDPN VPQSLENIVL
Limo_WP_003733096.1                                DGESAVSIAI KHLQADIPSA ----REQNPE IPQSLENIII
Listeria_multispecies_WP_003767439.1               DGESAVSIAI KHLQAEIPSA ----RAQNPE IPQSLENIII
Stpn_CJR66226.1                                    DGESAVSIAL KHLQAETPSA ----KRWNPS VPQSVENIIL
Stpn_COC44426.1                                    DGESAVSIAL KHLQTETPSA ----RKWNPS IPQSVENIIL
Stpn_CVM85423.1                                    EGESAVSVAL KHLQSEPPSV ----RRWNPS VPQSVENIIL
Stpn_CVM86034.1                                    TGDTPLAVAY QHVREEPPAP ----SDVDPD LPPAFDPVVL
Chtr_CQB85900.1                                    TGDSAVAIAY QHVSEVATPL ----STLVPG LPVMWDKICA
Chtr_CRH87370.1                                    QGDSAVAVAY QHVSEPPKVP ----SLIAPD IPDAIDRVVM
Chtr_CRH89418.1                                    TGDSAVAVAY QHVQQLPPTP ----SSITPD VPEALDRVVM
PsAe_WP_121124925.1                                ---------- ----QSTPSG ----QTLMP- ----------
Errh_WP_016357241.1                                RGETPVEIAM KHMREPFPSV ----MKFNPT LPNSIANIIA
Errh_WP_173446533.1                                HGKTPTEIAV KHLRQPIPYV ----RDFNPA IPQAVENIIL
Chtr_CRH67572.1                                    EGENAIQIAT HHVNDPIPMP ----STLVSW LPREIDDFIS
Chtr_CRH88717.1                                    IGESPVAVAY AHVNDPMPRL ----TQTAEW MPDDIDSFIC
PsAe_SQG56831.1                                    EGETPFAVAY QHVQEDPAKP SEFVSDLTPT AAVNVDAVVL
Codi_WP_003849886.1                                EGETPFAVAY QHVQEDPVKP SEYIADLSPT AAINVDAVVL
Chtr_CRH62988.1                                    DGDDAISVAL KQVNEQPVPP ----SARNPR VDQALERIIL
PsAe_SQG59153.1                                    SGDTQIAHAY SRLDRTVPAP ----SDVIDG IPPLFDALVA
Codi_WP_003852190.1                                SGDNQIAHAY ARLDSAVPAP ----SSMIAG IPPLIDALVA
Chtr_CRH93390.1                                    ---------- ---------- ---------- MPQALENVVL
Chtr_CPS17231.1                                    ---------- ---------- ---------- ----------
Chtr_CQB89058.1                                    ---------- ---------- ---------- ----------

                                                  281
StAu_newman_A6QGC0.1                               RATEKDKANR YKTIQEMKDD LSSVL----- ----------
Stau_aureus_CAA73980.1                             ---------- ---------- ---------- ----------
Ecoli_MQS28384                                     ---------- ---------- ---------- ----------
PsAe_MUK59325.1                                    ---------- ---------- ---------- ----------
Stau_SCT90678.1                                    RATEKDKANR YKTIQEMKDD LSSVL----- ----------
Stpn_Bacilli_multispecies_WP_048762376.1           KATEKNPKDR YQTIEEMYND LSSVL----- ----------
PsAe_WP_150019979.1                                ---------- ---------- ---------- ----------
PsAe_WP_121410777.1                                ---------- ---------- ---------- ----------
Enfu_VFA68308.1                                    KATAKKLTDR YKTTYEMGRD LSTAL----- ----------
Stpn_WP_160544498.1                                KATAKKLGDR YVSTTDMYQD LGTSL----- ----------
Stpn_Streptococcus_multispecies_WP_049527645.1     RATAKKLENR YNSTLEMSRD LVTSL----- ----------
PsAe_RUC36476.1                                    HATAKDPSDR YKTANEMAED LYTSL----- ----------
Enfu_WP_016624302.1                                RATAKEPADR YKTADEMSDD LATAL----- ----------
Limo_WP_003733096.1                                KATAKDPFLR YQNAEEMEKD LQTCL----- ----------
Listeria_multispecies_WP_003767439.1               KATAKDPFLR YQNAEEMEKD LQTCL----- ----------
Stpn_CJR66226.1                                    KATAKDPFHR YETAEDMEAD IKTAF----- ----------
Stpn_COC44426.1                                    KATAKDPFHR YESAEDMEAD IRTAF----- ----------
Stpn_CVM85423.1                                    KAMAKDPFYR YEDASEMQKD LKTAF----- ----------
Stpn_CVM86034.1                                    RALRKDPEDR FPTGTAFLAA LEEAA----- ----------
Chtr_CQB85900.1                                    KAMAKDRQNR YATAAEFKND ILAFM----- ----------
Chtr_CRH87370.1                                    KSLAKKREDR YQTASEMRTD LLAAL----- ----------
Chtr_CRH89418.1                                    KALAKDRTDR YTSAASMLAD LMRAS----- ----------
PsAe_WP_121124925.1                                ---------- ---------- ---------- ----------
Errh_WP_016357241.1                                RATHKNRTHR YANMKEFVED LDTCL----- ----------
Errh_WP_173446533.1                                KATAKDPKDR YSSAYAMLED LQNCM----- ----------
Chtr_CRH67572.1                                    ALAATNPDER PTDAGEALEL LARVEIGLPE EILTRRADVA
Chtr_CRH88717.1                                    TLTAKDPSKR PLNASAALDM LTNVITHLDD ADQMRRIPVF
PsAe_SQG56831.1                                    TAMAKHPGDR YQTAMDMGAD LERLA----- ----------
Codi_WP_003849886.1                                TAMSKHPGDR YQTAQEMCAD LERLE----- ----------
Chtr_CRH62988.1                                    KCMQKDPKNR FQTAEEVFKV LRDYL----- ----------
PsAe_SQG59153.1                                    SATTLSPHDR FTDADEFLTA LDDVA----- ----------
Codi_WP_003852190.1                                SATALRPEER FADAAEFLNA LDDVA----- ----------
Chtr_CRH93390.1                                    KATAKKITDR YKTVAEMYAD LASSL----- ----------
Chtr_CPS17231.1                                    ---------- ---------- ---------- ----------
Chtr_CQB89058.1                                    ---------- ---------- ---------- ----------
```

```
                                               321
StAu_newman_A6QGC0.1                           --------HE NRANEDVYEL DK-------- ----------
Stau_aureus_CAA73980.1                         ---------- ---------- ---------- ----------
Ecoli_MQS28384                                 ---------- ---------- ---------- ----------
PsAe_MUK59325.1                                ---------- ---------- ---------- ----------
Stau_SCT90678.1                                --------HE NRANEDVYEL DK-------- ----------
Stpn_Bacilli_multispecies_WP_048762376.1       --------VT SRLNEEKHTR IS-------- ----------
PsAe_WP_150019979.1                            ---------- ---------- ---------- ----------
PsAe_WP_121410777.1                            ---------- ---------- ---------- ----------
Enfu_VFA68308.1                                --------SS TRHREPKLVF N--------- ----------
Stpn_WP_160544498.1                            --------DS RRARESKLVF Q--------- ----------
Stpn_Streptococcus_multispecies_WP_049527645.1 --------YP SHSRDAKVVF D--------- ----------
PsAe_RUC36476.1                                --------SA SRLNEPAWEP TA-------- ----------
Enfu_WP_016624302.1                            --------SP GRSNEALWMP AA-------- ----------
Limo_WP_003733096.1                            --------NK DRLNEPKYVF PT-------- ----------
Listeria_multispecies_WP_003767439.1           --------NK DRLNEPKYVF PTN------- ----------
Stpn_CJR66226.1                                --------DA DRLNEKRFTI QE-------- ----------
Stpn_COC44426.1                                --------DA GRLNEQKFSV QD-------- ----------
Stpn_CVM85423.1                                --------DP ARLKEKRFVI EE-------- ----------
Stpn_CVM86034.1                                --------AD PHAGPGRHTP PLDAFPPALV GAGGAVLGAA
Chtr_CQB85900.1                                --------NG GVPVAAAFNP LT-------- ----------
Chtr_CRH87370.1                                --------RG DAIHAPAIGT WE-------- ----------
Chtr_CRH89418.1                                --------RG GHVNAPAVAV WA-------- ----------
PsAe_WP_121124925.1                            ---------- ---------- ---------- ----------
Errh_WP_016357241.1                            --------LE SRADEPLWEA TMES------ ------DDGT
Errh_WP_173446533.1                            --------NW EMRNVERISF TT-------- ----------
Chtr_CRH67572.1                                P-----KVAS SAGETATWE- ---------- ----------
Chtr_CRH88717.1                                PHKPII--EK TEITSSHTSI SSTS------ ----------
PsAe_SQG56831.1                                --------RH AVTEAARHYV SPASL----- ----------
Codi_WP_003849886.1                            --------RN AVTDAARHYV TPTSF----- ----------
Chtr_CRH62988.1                                ---------A GKMNE----- ---------- ----------
PsAe_SQG59153.1                                --------AE LQLPAFKVPV PANAA----- -AHRASENIN
Codi_WP_003852190.1                            --------QE LRLPEFRVPV PANAA----- -AYRSNEHLT
Chtr_CRH93390.1                                --------SS SRKNEKKVEL SD-------- ----------
Chtr_CPS17231.1                                ---------- ---------- ---------- ----------
Chtr_CQB89058.1                                ---------- ---------- ---------- ----------

                                               361
StAu_newman_A6QGC0.1                           -------MKT IAVPLKKEDL AKHISEHKSN QP--------
Stau_aureus_CAA73980.1                         ---------- ---------- ---------- ----------
Ecoli_MQS28384                                 ---------- ---------- ---------- ----------
PsAe_MUK59325.1                                ---------- ---------- ---------- ----------
Stau_SCT90678.1                                -------MKT IAVPLKKEDL AKHISEHKSN QP--------
Stpn_Bacilli_multispecies_WP_048762376.1       -------DTT QTVPIDKKEI KNKLDEENHK KD--------
PsAe_WP_150019979.1                            ---------- -VEPIFDDET AELN------ ----------
PsAe_WP_121410777.1                            ---------- ---------- ---------- ----------
Enfu_VFA68308.1                                -----DTEST KTLPKVTSTV SSLTTEQLLR N---------
Stpn_WP_160544498.1                            -----DTTDT KTLPKIEPSP VKEAPKAVVA ----------
Stpn_Streptococcus_multispecies_WP_049527645.1 -----DMTDT KTLPKVTPVP SVSSEKKATA K---------
PsAe_RUC36476.1                                -----LLGET KVLTPIPEDI AEPEETTPVE ----------
Enfu_WP_016624302.1                            -----MHNET KAIEPIDEEA LHQTESMHAE ----------
Limo_WP_003733096.1                            -----DDGDT KAIPIIATKD TMQNLDKTIV ----------
Listeria_multispecies_WP_003767439.1           ----DDDGDT KTIPIIATKE AMQNLDKTIV ----------
Stpn_CJR66226.1                                -----DEEMT KAIPIIKDEE LAKAAGEKE- ----------
Stpn_COC44426.1                                -----DEEMT KAIPVITDGA KAASQAETP- ----------
Stpn_CVM85423.1                                -----DHEAT KAIPIIKDHQ VDQDNEKTAV ----------
Stpn_CVM86034.1                                AAEHGADVPM AAVPLTRPTP SVPAEPDAAQ PQAA---NHA
Chtr_CQB85900.1                                -----DLANM RARKQAEQNA ATQAMNNAD- ----------
Chtr_CRH87370.1                                -----TYVSP SPLPPSAAAT Q--------- ----------
Chtr_CRH89418.1                                -----DTVPM AAANVANSQP TQTLPPAVAS ----------
PsAe_WP_121124925.1                            ---------- ---------- ---------- ----------
Errh_WP_016357241.1                            KLIEKLNGVT ETLP------ ---------- ----------
Errh_WP_173446533.1                            ---------- ---PNIAKVD V--------- ----------
Chtr_CRH67572.1                                -----HLGVT SSLPPTGHST S--------- ----------
Chtr_CRH88717.1                                ---HSAQSHT STIPPKRTDE LEIVTTSEIF PTQQ------
PsAe_SQG56831.1                                --ISQEPNPT TIVPITQLDQ PGPASAA--- ----------
Codi_WP_003849886.1                            --ATQDPAST TVVPVTQVAE LDHAEAGAGI GA--------
Chtr_CRH62988.1                                -----VNNAT AQMPVMATNA VSPLNSTTAL PH--------
PsAe_SQG59153.1                                GALSATDLLT TDIPRDATEL IDDAPGHGLF PHSQYPTTHE
Codi_WP_003852190.1                            VHFTTSDLMT TDLPRETEGL ---------- ----------
Chtr_CRH93390.1                                -----NKVDT KTLPKLSQST TEVKPVKAKS ----------
Chtr_CPS17231.1                                ---------- ---------- ---------- ----------
Chtr_CQB89058.1                                ---------- ---------- ---------- ----------
```

```
                                                401
StAu_newman_A6QGC0.1                            ---------K RETTQVPIVN G--------- ----------
Stau_aureus_CAA73980.1                          ---------- ---------- ---------- ----------
Ecoli_MQS28384                                  ---------- ---------- ---------- ----------
PsAe_MUK59325.1                                 ---------- ---------- ---------- ----------
Stau_SCT90678.1                                 ---------K RETTQVPIVN G--------- ----------
Stpn_Bacilli_multispecies_WP_048762376.1        ---------I AQTMQIPIIN ---------- ----------
PsAe_WP_150019979.1                             ---------- ---------- ---------- ----------
PsAe_WP_121410777.1                             ---------- ---------- ---------- ----------
Enfu_VFA68308.1                                 ------QK-Q AKT------- ---------- ----------
Stpn_WP_160544498.1                             ------PD-S VEV------- ---------- ----------
Stpn_Streptococcus_multispecies_WP_049527645.1  ------SS-E SKQ------- ---------- ----------
PsAe_RUC36476.1                                 -----VPE-- ---------- ---------- ----------
Enfu_WP_016624302.1                             ------PE-- ---------- ---------- ----------
Limo_WP_003733096.1                             ------PEGK VAA------- ---------- ----------
Listeria_multispecies_WP_003767439.1            ------PEGK VAA------- ---------- ----------
Stpn_CJR66226.1                                 ---------- ---------- ---------- ----------
Stpn_COC44426.1                                 ---------- ---------- ---------- ----------
Stpn_CVM85423.1                                 ---------- ---------- ---------- ----------
Stpn_CVM86034.1                                 VAGAGLPG-A ASAAQLAAAA AMDGPRPVTV GQPVKPGAAA
Chtr_CQB85900.1                                 ---------T ASTQSFGAIT G--------- --------QF
Chtr_CRH87370.1                                 LN-PVIPA-T AATNSYP--- ---------- ----------
Chtr_CRH89418.1                                 ------PS-N ATT------- ---------- ----------
PsAe_WP_121124925.1                             ---------- ---------- ---------- ----------
Errh_WP_016357241.1                             ---------- ---------- ---------- ----------
Errh_WP_173446533.1                             ------NDGH VEV------- ---------- ----------
Chtr_CRH67572.1                                 ---------- ---------- ---------- ----------
Chtr_CRH88717.1                                 LA-ALTPH-T ADDSQTNNDT E--------- ----------
PsAe_SQG56831.1                                 ------PH-S EVD------- ---------- ----------
Codi_WP_003849886.1                             ---GVVPA-G AVA------- ---------- ----------
Chtr_CRH62988.1                                 -----IAN-G ATQ------- ---------- ----------
PsAe_SQG59153.1                                 TSVFPATESA TETQIFPHEG LID------- AAPPAPSIAP
Codi_WP_003852190.1                             ---FPIEDAS THTRAFQPIT PQP------- ATPIGPVAQR
Chtr_CRH93390.1                                 ---------- ---------- ---------- ----------
Chtr_CPS17231.1                                 ---------- ---------- ---------- ----------
Chtr_CQB89058.1                                 ---------- ---------- ---------- ----------

                                                441
StAu_newman_A6QGC0.1                            -P-AHHQQFQ KPEGTVYEPK ------PKKK --STRKI---
Stau_aureus_CAA73980.1                          ---------- ---------- ---------- ----------
Ecoli_MQS28384                                  ---------- ---------- ---------- ----------
PsAe_MUK59325.1                                 ---------- ---------- ---------- ----------
Stau_SCT90678.1                                 -P-AHHQQFQ KPEGTVYEPK ------PKKK --STRKI---
Stpn_Bacilli_multispecies_WP_048762376.1        -----HHKFQ ASENNVYTPQ ------RKKR --SKKKK---
PsAe_WP_150019979.1                             ---------- ---------- ---------- ----------
PsAe_WP_121410777.1                             ---------- ---------- ---------- ----------
Enfu_VFA68308.1                                 -----TEKIT PDSASNDKTK ------SKK- ---KASHR--
Stpn_WP_160544498.1                             -----PKEKE ASKKEASTPP ------SSK- ---KSKKP--
Stpn_Streptococcus_multispecies_WP_049527645.1  -----AVSKQ PRKNSTLAKN ------KKK- ---KKKKS--
PsAe_RUC36476.1                                 --------DI ADDILAEQPP ------KKN- ---RKKL--W
Enfu_WP_016624302.1                             ------ELEA ADETPAEKQP ------KKK- ---KRKT--W
Limo_WP_003733096.1                             ------AEVV PEEKKDKKKK ------KMS- ---KKKK--I
Listeria_multispecies_WP_003767439.1            ------EEVA VDDKKGKKKK ------KMS- ---KKKK--V
Stpn_CJR66226.1                                 ---------- AEVTTAQENK ------TKKN --GKRKK--W
Stpn_COC44426.1                                 ---------- GETDDKEQQP ------AKK- ---KKRK--W
Stpn_CVM85423.1                                 -------HPV QKPKKKEKQK ------PKK- ---KRKK--W
Stpn_CVM86034.1                                 TP--RPEPQG DTPAPATATH ------RRV- ---RRRLRRR
Chtr_CQB85900.1                                 EPYTQNTGYN TRAAQLAANR ------AKR- ---SKRI---
Chtr_CRH87370.1                                 ----FTSQTA TDITPAIEEE ------EET- ---QSKK--W
Chtr_CRH89418.1                                 -------TFA PVATQELEEE ------PEK- ---KSKKG--
PsAe_WP_121124925.1                             ---------- ---------- ---------- ---NQKL---
Errh_WP_016357241.1                             ---------- ------NQSK ------PKK- ---KRKM---
Errh_WP_173446533.1                             -------EYS DEPIETEE-- ---------- ---KRKFPWF
Chtr_CRH67572.1                                 -----RTVVQ AAGVQGAMHS ------PKK- ---NRRS---
Chtr_CRH88717.1                                 ----NSIQTG TYVTTAEQTH ------ESRK GGTRSKK---
PsAe_SQG56831.1                                 -----GYEHS AFAGPAHAAE ------EERS G-MSKAMK--
Codi_WP_003849886.1                             -----GSAAV AGAGGAHAAP ------RSS- ---NRGLR--
Chtr_CRH62988.1                                 -----NRKSA THRAQLRAQE ------DEK- ---RHKNKVI
PsAe_SQG59153.1                                 DPYAAAAPAA PETIPPGPVE EPPVERPIT- --NRSKTS--
Codi_WP_003852190.1                             KPDYSPEPVA PQHAPAATHE ------PVT- --NRSKIS--
Chtr_CRH93390.1                                 -----PAKNN AKQAEAEKKT ------DKI- ---KRRMRTR
Chtr_CPS17231.1                                 ---------- ---------- ---------- ----------
Chtr_CQB89058.1                                 ---------- ---------- ---------- ----------
```

```
                                            481
StAu_newman_A6QGC0.1                        --VLLSLIFS LLM---IALV SFVA-----M AMF-----GN
Stau_aureus_CAA73980.1                      ---------- ---------- ---------- ----------
Ecoli_MQS28384                              ---------- ---------- ---------- ----------
PsAe_MUK59325.1                             ---------- ---------- ---------- ----------
Stau_SCT90678.1                             --VLLSLIFS LLM---IALV SFVA-----M AMF-----GN
Stpn_Bacilli_multispecies_WP_048762376.1    --VAIIFILA LLL---LSLI GFIA-----W GML-----GD
PsAe_WP_150019979.1                         ---------- ---------- ---------- ----------
PsAe_WP_121410777.1                         ---------- ---------- ---------- --------SD
Enfu_VFA68308.1                             --LLGTIMKL FFA---LCVV GIIVFAY--K ILV-----SP
Stpn_WP_160544498.1                         --FWQRLFRF FIL---ALVL LLAGFAF--F FMS----SSN
Stpn_Streptococcus_multispecies_WP_049527645.1 --FFSTSLKV FLG---LVFI GIIIFAY--L VFT-----NP
PsAe_RUC36476.1                             IGLAIAALIA LAI----GGL AFAMS----- --------GG
Enfu_WP_016624302.1                         LWILIAALVA LVA---GAAV YFAAT----- --------RN
Limo_WP_003733096.1                         ALIVSSVIII FII---GILL L--------W LLG----KSP
Listeria_multispecies_WP_003767439.1        ILIVSSVIVL FII---GILL L--------W LLG----KGP
Stpn_CJR66226.1                             PWVLLTICLV FIT---AGIL AVTVFPSL-- -------FMP
Stpn_COC44426.1                             PWVLLAVCFI FIM---AAVL AVTVFPSL-- -------FMP
Stpn_CVM85423.1                             PWIVASILFV LTA---ATIL AITVFPGL-- -------LFP
Stpn_CVM86034.1                             TLLAAGAVLI GLA--LVLAP WLAAA----- --------LR
Chtr_CQB85900.1                             --VITSVISA VVCAIVVAGI LLVLNRS--- -------KLA
Chtr_CRH87370.1                             LWVTLAVLGV IVA---MAGL FFGLKAA--G LLGTDPSLEI
Chtr_CRH89418.1                             --IIAALVVV ALL--LMSGI WYALSHAS-- -------SDK
PsAe_WP_121124925.1                         --ILI----- ---------- ---------- --------TN
Errh_WP_016357241.1                         --IVGASLLA LVI--IIFSI WMIFAPK--- --------KP
Errh_WP_173446533.1                         TWIIRTIVIL IVC--LILLL GLVAGHVI-Q VDG-----LL
Chtr_CRH67572.1                             --FIGKFLAI IAF---VAII GGVGGWWWWN EYG-----PG
Chtr_CRH88717.1                             --LITALLLI ITL---IAAL SSGIYWW--F FFG-----PG
PsAe_SQG56831.1                             --AALAVLAV LVL---GVGG AFAYD----- FVSN-SSKNR
Codi_WP_003849886.1                         --ILAAILAV LVL---AVGA GFAID----H FGGG-PFSQR
Chtr_CRH62988.1                             AGVLGGTLVL IAI--IVALV S--------Y FMG-----QG
PsAe_SQG59153.1                             --FIIWLVLV LVV---TASV AIGG-----W WFG-----SG
Codi_WP_003852190.1                         --FVLWLIAV ITL---ATAV ALGA-----W WFG-----SG
Chtr_CRH93390.1                             YKVLIGAIFL LAA----AFV T--------I LLS-----TP
Chtr_CPS17231.1                             ---------- ---------- ---------- ----------
Chtr_CQB89058.1                             --ILASVIIV LLA-----LL WMNLFTR--- --------HG

                                            521
StAu_newman_A6QGC0.1                        KYEETPD-VI GKSVKEAEQI FNKNNLKLGK -ISRSYSDKY
Stau_aureus_CAA73980.1                      ---------- ---------- ---------- ----------
Ecoli_MQS28384                              ---------- ---------- ---------- ----------
PsAe_MUK59325.1                             ---------- ---------- --KNNLKLGK -ISRSYSDKY
Stau_SCT90678.1                             KYEETPD-VI GKSVKEAEQI FNKNNLKLGK -ISRSYSDKY
Stpn_Bacilli_multispecies_WP_048762376.1    KYAEMPD-LT GKTEKEAEKV LKASHLEIGH -ISREYNDDY
PsAe_WP_150019979.1                         KKVEVPD-VI GKELGEAYAI LDEAGLNYLS DNS-------
PsAe_WP_121410777.1                         GSVTLPN-FT GFTYGEVRDW LHKAGLAFKP ----------
Enfu_VFA68308.1                             TTIRVPD-VS NKTVAQAKMT LENSGLKVGA -IRNIESDSV
Stpn_WP_160544498.1                         DSATVPD-VS GQTIQEAQAT LEEAGLTVGA -VSRKESDSV
Stpn_Streptococcus_multispecies_WP_049527645.1 DSTQVPN-VV GQELSTAQTK IEGAGFKVGE -VKEVEDDSV
PsAe_RUC36476.1                             KDVEVPD-VT NETKADASQA LQSAGLKVDS ETKKIPDDKI
Enfu_WP_016624302.1                         SDVVVPD-VS GLTESEARTK LEAVNLKVAS ETQEIADEEI
Limo_WP_003733096.1                         DEVAVPD-VS GKTEDQAVAL LQKEGFVIGK -TAEKNSDEV
Listeria_multispecies_WP_003767439.1        DEVAVPD-VS GKTEDQAIAL LQKDGFVIGK -TVEKNSDDV
Stpn_CJR66226.1                             KDVKIPD-VS GMEYEKAAGL LEKEGLQVDS EVLEISDEKI
Stpn_COC44426.1                             KDVSVPD-VR GMKYEKAEAL LEKNGLRADP DITDIEDEKI
Stpn_CVM85423.1                             KDVEVTD-VS GMTVEKAEDT LKKAGFTVAS EPIDIADEKI
Stpn_CVM86034.1                             TTVEVPA-VV GLQQDEAVAA LEDAGLVAVV --SSAYMADA
Chtr_CQB85900.1                             GEVVVPTFTE TTTQDAAREK LRALGLIPDI --LEDDKSSQ
Chtr_CRH87370.1                             TQVDVPD-MK GMDEDAARKA LTEAGLKIKV -GDAVNDEKI
Chtr_CRH89418.1                             EMVAVPDNLI GMTQAEAKSA VESVGLVFAI SNETVASDEI
PsAe_WP_121124925.1                         GTNYMPD-LT GWSKSDVTKF GDLLGLTVEF ----------
Errh_WP_016357241.1                         SDIEIPD-LA GLSVSEAKEH LANLGLNYAP SYLYEISDKY
Errh_WP_173446533.1                         GYQTIPN-VV NLSTSQAKKK LKAKNFTHIT -IKKVLSDSV
Chtr_CRH67572.1                             SYLTMPQ-TA GRSSQAVKSE LAALGLGVIE --EEAFSDTV
Chtr_CRH88717.1                             LRVSIPQ-VA GMSVAEAEKT LTTVGFQTTT --AHEYSDTV
PsAe_SQG56831.1                             SLVNIPK-LE KSSQQDAVNQ LEQLGLQVNV --IEEANPDV
Codi_WP_003849886.1                         STVTIPK-LQ NSTQQDAVNQ LEKLGLQVNV --IEEPNPDI
Chtr_CRH62988.1                             ATQEVPN-LL NLTQQEALMK IDNSNFFQRG TVKEEFSSTV
PsAe_SQG59153.1                             RYGEVPQ-VL GMSQIEATAL AQDAGFSTTT --SPVYSDDI
Codi_WP_003852190.1                         RYGEVPQ-VL GLDQVQAVAV TEESGFGAVT --EPVYSDDV
Chtr_CRH93390.1                             ATVKVPD-VA GLTRDQAVDK IESAGLKIGQ -VKEEASSDV
Chtr_CPS17231.1                             -MVTLKD-LS NYSLKSAQDY AKEHGLTLQI --NQEYSDDV
Chtr_CQB89058.1                             SSVEIPD-IY GLPESEAEQL LDKAHLRYEI -IDSVYTTEV
```

```
                                                561
StAu_newman_A6QGC0.1                            PENEIIKTTP NTGERVERGD SVDVVISKGP E---KVKMPN
Stau_aureus_CAA73980.1                          ---------- ---------- ---------- ----------
Ecoli_MQS28384                                  ---------- ---------- ---------- ----------
PsAe_MUK59325.1                                 PENEIIKTTP NTGERVERGD SVDVVISKGP E---NVKMPN
Stau_SCT90678.1                                 PENEIIKTTP NTGERVERGD SVDVVISKGP E---KVKMPN
Stpn_Bacilli_multispecies_WP_048762376.1        PENKIINSNP KAGERVNQQE KVDVVLSKGP E---KINMPN
PsAe_WP_150019979.1                             --GSIVKD-- ---------- ---------- ----------
PsAe_WP_121410777.1                             ---------- ---------- ---------- ---------D
Enfu_VFA68308.1                                 SEGLVVKTDP AAGRSRREGA KVNLYIATPN K---SFTLGN
Stpn_WP_160544498.1                             EEGKVISTTP KSGTTQKEGS EVDLLVSKGS K---RFVMTD
Stpn_Streptococcus_multispecies_WP_049527645.1  DTGKVIKTDP TAGTTRKEGS SIDIYVSSGS K---GFALKD
PsAe_RUC36476.1                                 EEGKVVKTDP EAKSSVKKGR SVTLYISSGT E---KIEMAD
Enfu_WP_016624302.1                             EEGNVVKTDP AATTTVKENR EVTLYISSGS K---AIELKD
Limo_WP_003733096.1                             DEGKVINTDP EAGEMKEKGT KINLFVSIGS K---KITMDD
Listeria_multispecies_WP_003767439.1            EEGKVINTDP SAGEMKEKGT KINLFVSIGS K---KITMED
Stpn_CJR66226.1                                 EEGLMVKTDP KADTTVKEGA TVTLYKSTGK A---KTEIGD
Stpn_COC44426.1                                 EEELMVKTDP KAGSTVKEGS SVKLYKSIGK P---KTQLID
Stpn_CVM85423.1                                 EKGLVVKTDP KIGTKVKEGT EIQLYQSTGK E---KTELPD
Stpn_CVM86034.1                                 PDGTVTQSDP PTGTEVPRGA KVRIRVSRGE E---GIALSD
Chtr_CQB85900.1                                 PEGTFTKQLP KGGAKVSSGS RVSVWFSVGP Q---STKIPD
Chtr_CRH87370.1                                 PEGQFVSSDP AIGTSVDKGS IVTVHFSAGI A---NVKVPD
Chtr_CRH89418.1                                 EEGKVAKTDP PSGNQVEVGS TVTAKLSSGA E---SVVIPD
PsAe_WP_121124925.1                             ---------- ---------- ---------- ----------
Errh_WP_016357241.1                             EDGKLIGTKP EKGTKVLKGD QIKLIVSQGK ----IYTVED
Errh_WP_173446533.1                             DKGQVISCNY DAGQSVYHGN TIVLKVSKGP G----YLVED
Chtr_CRH67572.1                                 QSGIVISSDP DGGQPVHKNA EVRVTTSKGI D---MRAVPD
Chtr_CRH88717.1                                 DKDTVIASTP EGGTTAHPSQ IITLRISDGV E---YLNVPN
PsAe_SQG56831.1                                 PRGKVIRTNP TSGSSVQKNT TVTVVVSSGK E---VTEVPD
Codi_WP_003849886.1                             PRGKVIRTNP TDGSNVQRNS TVRLTISSGK E---ITEVPD
Chtr_CRH62988.1                                 EKGKVIDQDP DAGHKVAKGT QINIVVSKGA EPAADVTVPD
PsAe_SQG59153.1                                 PADLVAGAIP EVGHRAVKGN EITLLVSQGK P-----TVPS
Codi_WP_003852190.1                             PKNAIAGTKP DQGQRAVKGD DITLLVSQGR P-----TVPD
Chtr_CRH93390.1                                 EAGKVIRTDP AAKTSRREGS AVDLFIAV-E E---AVTVPD
Chtr_CPS17231.1                                 EKGLVISMEP GPGTKVERGS TVTIKISKGP KEDKETTVTK
Chtr_CQB89058.1                                 PKGAVYDLTP KAGSKVKAGR IIFITLNAYC P--RNGIIPS

                                                601
StAu_newman_A6QGC0.1                            -VIG--LPKE EALQKL-KSL GLK--DVTIE KVYNN-----
Stau_aureus_CAA73980.1                          ---------- --MQKL-KSL GLK--DVTIE KVYNN-----
Ecoli_MQS28384                                  ---------- ---------- ---------- -VYNN-----
PsAe_MUK59325.1                                 -VIG--LPKE QALQKL-KSL GLK--DVKIE KVYNN-----
Stau_SCT90678.1                                 -VIG--LPKE EALQKL-KSL GLK--DVTIE KVYNN-----
Stpn_Bacilli_multispecies_WP_048762376.1        -VIG--IKKE DAIKKL-KDH KLN--HVTIN QEYNS-----
PsAe_WP_150019979.1                             ---------- ---------- ---------- ----------
PsAe_WP_121410777.1                             ---------- ---------- ---------- ----------
Enfu_VFA68308.1                                 -YKE--HNYK DILKDL-QGK GVKKSLIKVK RKINN-----
Stpn_WP_160544498.1                             -YTG--MSYR DAVSDLTENY GLSKNQIVKK EVTNN-----
Stpn_Streptococcus_multispecies_WP_049527645.1  -YKG--KNYK DAIEDLTSNY GVSEDQIDIQ HVEDD-----
PsAe_RUC36476.1                                 -YTN--ESYE SAVEAL-KKL GFSEDQITTK KEY-------
Enfu_WP_016624302.1                             -YTD--MSYE EVESEL-LSL GFSASQIKKT TEFNS-----
Limo_WP_003733096.1                             -YTG--RSYT DTKALL-EEQ GFK--NISSE EAYSS-----
Listeria_multispecies_WP_003767439.1            -YTG--RSYS ETKTLL-EQQ GFS--NISAE EAYSS-----
Stpn_CJR66226.1                                 -VTG--QTVD QAKKAL-KDQ GFN--HVTVN EVND------
Stpn_COC44426.1                                 -VKG--RQIG DAKKAL-KEK GFK--HVNVK EEND------
Stpn_CVM85423.1                                 -VTG--EKVE KAKERL-EKK GFK--QVVVE EVNDN-----
Stpn_CVM86034.1                                 SLHG--LSED AARREL-ERL GLR--VSSVQ YQDDG-----
Chtr_CQB85900.1                                 -VTG--KSQD VARKAL-ERA GFK--ISNVR VEDST-----
Chtr_CRH87370.1                                 -VTGGTSTQE EARKQL-EEA GLR--VGNVE TTDEP-----
Chtr_CRH89418.1                                 NLVG--MSPD AAKQAI-EAL GLVY-EQTAD PVASA-----
PsAe_WP_121124925.1                             ---------- ---------- ---------- ----------
Errh_WP_016357241.1                             -FTG--KSVN DVKAIL-SDK NIN---IKTT SEYSS-----
Errh_WP_173446533.1                             -FTG--QTLE EAEYTL-DKE GVKL-NIEVK YKNAA-----
Chtr_CRH67572.1                                 -LVG--KNKS EIETLL-IDA GLA--VGTIN EEYSE-----
Chtr_CRH88717.1                                 -VVG--KTAD EAQHIL-TQA RFA---ASAS EDWSN-----
PsAe_SQG56831.1                                 -LAR--KNTA DAAKIL-EEA GLQL-DSTVR EESSD-----
Codi_WP_003849886.1                             -LSG--KNTA DAVKIL-EAA GLLL-DPTVR EDSSD-----
Chtr_CRH62988.1                                 -LKG--KSPS EAEAIL-SQL GLK--SQAGD SVNSD-----
PsAe_SQG59153.1                                 -IPS-SHDTA EVRALL-EER SLS--YSEAP PEYSD-----
Codi_WP_003852190.1                             -VPQ-SRSAS EYRRLL-EER TLE--YRESA PEFSD-----
Chtr_CRH93390.1                                 -VSG--SDLE TAKAKL-QEL GFK--VGEVK YKNSE-----
Chtr_CPS17231.1                                 TFTV------ ---------- ---------- ----------
Chtr_CQB89058.1                                 -LID--VSER QARARL-ISL GFE--NITPS YVAGPFDGLV
```

```
                                              641
StAu_newman_A6QGC0.1                          ---QAPKGYI ANQSVT-ANT EIAIHDSN-- IKLYESLGIK
Stau_aureus_CAA73980.1                        ---QAPKGYI ANQSVT-ANT EIAIHDSN-- IKLYESLGIK
Ecoli_MQS28384                                ---QAPKGYI ANQRVT-ANT EIAIHDSN-- IKLYESLGIK
PsAe_MUK59325.1                               ---QAPKGYI ANQSVT-ANT EIAIHDSN-- IKLYESLGIK
Stau_SCT90678.1                               ---QAPKGYI ANQSVT-ANT EIAIHDSN-- IKLYESLGIK
Stpn_Bacilli_multispecies_WP_048762376.1      ---QMPKGYI FKQNIN-PNE SVKINDHH-- IVLTESLGVK
PsAe_WP_150019979.1                           ---------- ---------- ---------- ----------
PsAe_WP_121410777.1                           ---------- ---------- ---------- ----------
Enfu_VFA68308.1                               ---DYTTGTI LAQSLP-EGT SFNPDGNK-K LTLTVAV-ND
Stpn_WP_160544498.1                           ---QYEPGEI ISQTPE-AGE DFNPSGND-K IIFEVAT-AS
Stpn_Streptococcus_multispecies_WP_049527645.1 ---SAEEGEI LSQSPG-KNK SFNPKDSKAK IKFRVAT-PK
PsAe_RUC36476.1                               ---------- ---------- ---------- ----------
Enfu_WP_016624302.1                           ---DVEEGNI ISQDPG-KGS KVEPDSDT-- ISFVVSDGPQ
Limo_WP_003733096.1                           ---EIEKGLI ISQTPT-QGT EVVAKSTD-- VKFVVSKGAE
Listeria_multispecies_WP_003767439.1          ---EVDKGMI ISQTPT-QGT EVVAKSTD-- VKFVVSKGAE
Stpn_CJR66226.1                               ---EKNAGTV IDQNPS-AGT ELVPSEDQ-- VKLTVSIGPE
Stpn_COC44426.1                               ---DSEAGTV IDQDPS-AGT DMVASDDE-- VNLTVSLGPA
Stpn_CVM85423.1                               ---DTESGIV MEQKPS-ANT ELVAADEE-- VTLTVSIGPA
Stpn_CVM86034.1                               ---RLERGLL ARTDPA-MGT HV-PPGSS-- VVLHLSSGM-
Chtr_CQB85900.1                               ---EVKKNHV TRTDPS-ADS FA-DKGSM-- VTLYISSGLT
Chtr_CRH87370.1                               ---GIAQGMV VSTDPA-PGT VI-PKQTP-- VTLYVASGE-
Chtr_CRH89418.1                               ---DVEEGKV AQVNPS-VGS KV-KKGST-- VKVSLSSGPK
PsAe_WP_121124925.1                           ---------- ---------- ---------- ----------
Errh_WP_016357241.1                           ---SVPTGHT IRQEGLMPGD KIKPEQRY-D IVLVVSSDKE
Errh_WP_173446533.1                           ---DTNPGVI LSQKKLKAGT RIDPNANE-T IQFVVSQNPT
Chtr_CRH67572.1                               ---EVAKDIV ISQSLD-PDT SI-PHDTK-- IDVVVSKGRE
Chtr_CRH88717.1                               ---DVPKGTV ISQTPE-AGE SI-PHDSS-- VTYIVSKGRE
PsAe_SQG56831.1                               ---SVPNGEI IEQTPS-AGS QV-SRGSK-- VVITVSTGVQ
Codi_WP_003849886.1                           ---TVPKGEI IEVSPA-AGS QV-SRGSK-- VSITVSTGVE
Chtr_CRH62988.1                               ---DVEVGKI AAQTPA-AGT TA-KAGDT-- ITYQLSKGPN
PsAe_SQG59153.1                               ---DVPEGKV VSLKPE-PGT TV-RVGSE-- VAVALSRGPA
Codi_WP_003852190.1                           ---SVPEGKI ARVVPG-SGN EV-AVGSA-- VSVALSQGPA
Chtr_CRH93390.1                               ---SVAEGNV ISTAPK-ANT SK-SKGST-- INLVVSSGVE
Chtr_CPS17231.1                               ---------- ---------- ---------- ----------
Chtr_CQB89058.1                               MGVQLPSGQT LA-----PGT RV-PLSTP-- LILLISVST-

                                              681
StAu_newman_A6QGC0.1                          QVYVEDFEHK SFSKAKKALE EKG--FKVES KEEY------
Stau_aureus_CAA73980.1                        QVYVEDFEHK SFSKAKKALE EKG--FKVES KEEY------
Ecoli_MQS28384                                QVYVEDFEHK SFSKAKKALE EKG--FKVES KEEY------
PsAe_MUK59325.1                               QVYVEDFEHK SFSKAKKALE EKG--FKVES KEEY------
Stau_SCT90678.1                               QVYVEDFEHK SFSKAKKALE EKG--FKVES KEEY------
Stpn_Bacilli_multispecies_WP_048762376.1      KVYVKDYENK NYQNAKKELE SRG--LKVQV KTTN------
PsAe_WP_150019979.1                           ---------- ---------- ---------- ----------
PsAe_WP_121410777.1                           ---------- ---------- --G------- ----------
Enfu_VFA68308.1                               PMIMPDVTGM TVGEVIETLT DLG--LDADN LVFYQMQNGV
Stpn_WP_160544498.1                           TVTIPTLVGY SYADAKNALL DLG--FKASQ IQTD------
Stpn_Streptococcus_multispecies_WP_049527645.1 TVTMPDVTGL TVSTAVQTLN RKS--ISSSS IEYHDYNTGA
PsAe_RUC36476.1                               ---------- ---------- ---------- ----------
Enfu_WP_016624302.1                           TVTLRNYTGE DYNAVYNELI AQG--FTDSM LSFS------
Limo_WP_003733096.1                           PITLKDLRGY TKTAVEDYAS PLG--LKVSS KEEN------
Listeria_multispecies_WP_003767439.1          PITLIDLRGY TKTAVEDYAS PLG--LKVSS KEEN------
Stpn_CJR66226.1                               DITLRDLKTY SKEAASGYLE DNG--LKLVE KEAY------
Stpn_COC44426.1                               DVTLRDLKTY SKEAASGYLE DNG--LQLVE KQAH------
Stpn_CVM85423.1                               DITLRDLTTY SKQAASNYLE DNG--LKLDE KEAY------
Stpn_CVM86034.1                               -VAVPDVVGM SAPDARRQLA LSAPELRVRI QDED------
Chtr_CQB85900.1                               KVP-DGLVGQ SKDVVTSELQ NLG--FTVNV VEEE------
Chtr_CRH87370.1                               -VKIDSYIGL SLSEAEKSLN ALK--IQFDT EQVS------
Chtr_CRH89418.1                               SVEVPDVTGM TQDQAREALA AKG--LTVGS VQVS------
PsAe_WP_121124925.1                           ---------- ---------- -KG------- ----------
Errh_WP_016357241.1                           LVLPGDLIGK SIADAKAQLE GLG--VEVTT SELPT-QNLS
Errh_WP_173446533.1                           IVIPESLIGM DVNEAKESLN DQG--IAVVL ----------
Chtr_CRH67572.1                               PLTVPDLMAM SSADAQAAIE ALG--LVASP SEEY------
Chtr_CRH88717.1                               PITIPKIGEL SGSDYEKALT DAG--FSVTK QEEF------
PsAe_SQG56831.1                               NVRVPVVTGM KWDQAEGNLT SLG--FVPDV RT--------
Codi_WP_003849886.1                           TVRVPVITGM KWDQAEGNLT SLG--FKPEV VR--------
Chtr_CRH62988.1                               EADVPNVVGK TEDEAQAALT AAG--FNVSV QREE------
PsAe_SQG59153.1                               PVNVPHVAEM SEESARDQLE KLG--LKVNI TEEF------
Codi_WP_003852190.1                           PVTIPNVSER PVDQARNELE KLG--FDVTI AKEF------
Chtr_CRH93390.1                               TVTVPDVAGI SQSVAKDALT AAG--LTVGT VTENA-----
Chtr_CPS17231.1                               -----NYVGD DLSKSEKPDN DKG------- ----------
Chtr_CQB89058.1                               ---------- ---------- ---------- ----------
```

```
                                                 721
StAu_newman_A6QGC0.1                             ---SDDID-E GDVISQSPK- GKSVD---EG STISFVV---
Stau_aureus_CAA73980.1                           ---SDDID-E GDVISQSPK- GKSVD---EG STISFVV---
Ecoli_MQS28384                                   ---SDDID-E GDVISQSPK- GKSVD---EG STISFVV---
PsAe_MUK59325.1                                  ---SDDID-E GDVISQSPK- GKSVD---EG STISFVV---
Stau_SCT90678.1                                  ---SDDID-E GDVISQSPK- GKSVD---EG STISFVV---
Stpn_Bacilli_multispecies_WP_048762376.1         ---SDTKD-K DIIISQSPK- QTEVN---EG STVELMV---
PsAe_WP_150019979.1                              ---------- -----QMPKP GAMVK---VN TTVLLY----
PsAe_WP_121410777.1                              ---------T GTAVSQDESG GTTVK---AG TAITVHF---
Enfu_VFA68308.1                                  YQAVVTPPSS SKIASQDPYY GGEVGL-RRG DKVKLYLLGS
Stpn_WP_160544498.1                              ---SDQPSSS ATVYAQYPYA GTSVEL-SSG ETVTLYL---
Stpn_Streptococcus_multispecies_WP_049527645.1   KLDKDKVPSS TEVLYQDPQA GTSVD----- GTVILYV---
PsAe_RUC36476.1                                  ---SDSVS-- ---------- ---------- ----------
Enfu_WP_016624302.1                              YVYDDNVA-S GSIVSQSVAA GVAID--PTR TTVSFEV---
Limo_WP_003733096.1                              ---SSTVE-K GQVISQSPSA GTAMN---SG DTIEIVI---
Listeria_multispecies_WP_003767439.1             ---SNSVE-K GQVISQSPSA GTAIN---AG DTIEIVI---
Stpn_CJR66226.1                                  ---SDDVP-E GQVVKQKPAA GTAVK---PG NEIEVTF---
Stpn_COC44426.1                                  ---SDDVP-E GQVMKQEPSA GTAVK---PG SNVEVTF---
Stpn_CVM85423.1                                  ---SDDVP-K GEVMKQEPGA GTAVK---PG DTVKITF---
Stpn_CVM86034.1                                  ---GATTD-T GTVVAQDPPA GVRVD---NR SSLTLTA---
Chtr_CQB85900.1                                  ---SDTAS-E GTVTKMNPSS GAAVK---PH SSVTVYV---
Chtr_CRH87370.1                                  ---SDEP--A GTIVGQNPGA GK-IA---YN SRVKLSI---
Chtr_CRH89418.1                                  ---DDPSQKE GLVISTNPAA GTSVN---KD TAVTLTL---
PsAe_WP_121124925.1                              ---------E GYVTKQSIAA ETEIT----- ----------
Errh_WP_016357241.1                              ISELNSLS-Y GTVIRSNPMP GSYYIQ-NSG NSVTLYY---
Errh_WP_173446533.1                              ---SGSGD-T GVVVNVSPSV GSTYTQEGSD SVVTLYYQ--
Chtr_CRH67572.1                                  ---SDSVA-E GSLISQTTAA GTTLY---KG DKVEYVV---
Chtr_CRH88717.1                                  ---SDSVP-E GAVISVDPAE GTQLY---RG DAVTLTI---
PsAe_SQG56831.1                                  ---VDAPEPA GTVVGV-SDE GTEIP---KG STVTVKV---
Codi_WP_003849886.1                              ---VDSVEPA GTVVAV-PDE GAEVP---KG SSVTVQI---
Chtr_CRH62988.1                                  ---SNKP--E GTVTKQSATG KLA-----RK STVTITV---
PsAe_SQG59153.1                                  ---NSEIR-G GDAIGTLPEA GTTLP---RG TTVTLRI---
Codi_WP_003852190.1                              ---SDTIP-G GDAIGTDPAA GTTLP---RG SSVTLKV---
Chtr_CRH93390.1                                  ---SDTVA-A GLVISTDPAA KAQVA---KG STVNLVI---
Chtr_CPS17231.1                                  ---SNNSE-- ---------- SESVN--GKG DHVQIYI---
Chtr_CQB89058.1                                  ---RDSIP-H GTLQPYDSIM GS------GG SAI----P--

                                                 761
StAu_newman_A6QGC0.1                             -----SKGKK SD-------- ---------- ----------
Stau_aureus_CAA73980.1                           -----SKGKK SD-------- ---------- ----------
Ecoli_MQS28384                                   -----SKGKK SD-------- ---------- ----------
PsAe_MUK59325.1                                  -----SKGKK SD-------- ---------- ----------
Stau_SCT90678.1                                  -----SKGKK A--------- ---------- ----------
Stpn_Bacilli_multispecies_WP_048762376.1         -----SKGKD KK-------- KDSKEDSDS- --------SD
PsAe_WP_150019979.1                              ---------- ---------- ---------- ----------
PsAe_WP_121410777.1                              -----SR--- ---------- ---------- ----------
Enfu_VFA68308.1                                  KTTNNSSSTP ID-------- ---------- ----------
Stpn_WP_160544498.1                              -----TTPEP SS-------- ---------- ----------
Stpn_Streptococcus_multispecies_WP_049527645.1   -----SVATA SS-------- ---------- ----------
PsAe_RUC36476.1                                  ---------- ---------- ---------- ----------
Enfu_WP_016624302.1                              -----SQGSQ PK-------- ---------- ----------
Limo_WP_003733096.1                              -----SAGPK EK-------- ---------- ----------
Listeria_multispecies_WP_003767439.1             -----SAGPK EK-------- ---------- ----------
Stpn_CJR66226.1                                  -----SLGPE KK-------- ---------- ----------
Stpn_COC44426.1                                  -----SLGPE EK-------- ---------- ----------
Stpn_CVM85423.1                                  -----SLGPK EK-------- ---------- ----------
Stpn_CVM86034.1                                  -----SSWIA PT-------- AEPSEPAPP- ----------
Chtr_CQB85900.1                                  -----SKGKP KV-------- ---------- ----------
Chtr_CRH87370.1                                  -----SSGKA PT-------- ---------- ----------
Chtr_CRH89418.1                                  -----SSGK- ---------- ---------- ----------
PsAe_WP_121124925.1                              ---------- ---------- ---------- ----------
Errh_WP_016357241.1                              ---------- ---------- ---------- ----------
Errh_WP_173446533.1                              ---------- ---------- ---------- ----------
Chtr_CRH67572.1                                  -----SKGPE TV-------- ---------- ----------
Chtr_CRH88717.1                                  -----SKGPE LL-------- ---------- ----------
PsAe_SQG56831.1                                  -----SNGQM ---------- ---------- ----------
Codi_WP_003849886.1                              -----SNGAM ---------- ---------- ----------
Chtr_CRH62988.1                                  -----AKAPS QV-------- ---------- ----------
PsAe_SQG59153.1                                  -----STAVE IPDVRGKSKQ EATSELAAAG IRVNSVTRSD
Codi_WP_003852190.1                              -----STSVE IPDVRGKSRH AAISALADAG ITVESISRSD
Chtr_CRH93390.1                                  -----SKGAA ---------- ---------- ----------
Chtr_CPS17231.1                                  ---------- ---------- ---------- ----------
Chtr_CQB89058.1                                  -----SDSTS HS-------- ---------- ----------
```

```
                                              801
StAu_newman_A6QGC0.1                          ---------- ---------- ---------- ----SSDVKT
Stau_aureus_CAA73980.1                        ---------- ---------- ---------- ----SSDVKT
Ecoli_MQS28384                                ---------- ---------- ---------- ----SSDVKT
PsAe_MUK59325.1                               ---------- ---------- ---------- ----SSDVKT
Stau_SCT90678.1                               ---------- ---------- ---------- ----------
Stpn_Bacilli_multispecies_WP_048762376.1      DKKDNH---- ---------- ---------- ----TTSVKN
PsAe_WP_150019979.1                           ---------- ---------- ---------- ----------
PsAe_WP_121410777.1                           ---------- ---------- ---------- ----------
Enfu_VFA68308.1                               ---------- ---------- ---------- ------SSAS
Stpn_WP_160544498.1                           ---------- ---------- ---------- ------TQPS
Stpn_Streptococcus_multispecies_WP_049527645.1 ---------- ---------- ---------- ------SLQS
PsAe_RUC36476.1                               ---------- ---------- ---------- ----------
Enfu_WP_016624302.1                           ---------- ---------- ---------- ------KLND
Limo_WP_003733096.1                           ---------- ---------- ---------- ------QVKE
Listeria_multispecies_WP_003767439.1          ---------- ---------- ---------- ------QVKE
Stpn_CJR66226.1                               ---------- ---------- ---------- ------PAKT
Stpn_COC44426.1                               ---------- ---------- ---------- ------PAKT
Stpn_CVM85423.1                               ---------- ---------- ---------- ------PAKT
Stpn_CVM86034.1                               ---------- ---------- ---------- ----SSEAPE
Chtr_CQB85900.1                               ---------- ---------- ---------- ------EVPR
Chtr_CRH87370.1                               ---------- ---------- ---------- ------PAPE
Chtr_CRH89418.1                               ---------- ---------- ---------- -----ITIPS
PsAe_WP_121124925.1                           ---------- ---------- ---------- ----------
Errh_WP_016357241.1                           ---------- ---------- ---------- ----------
Errh_WP_173446533.1                           ---------- ---------- ---------- ----------
Chtr_CRH67572.1                               ---------- ---------- ---------- ------EVPS
Chtr_CRH88717.1                               ---------- ---------- ---------- ------EVPN
PsAe_SQG56831.1                               ---------- ---------- ---------- -----FNVPN
Codi_WP_003849886.1                           ---------- ---------- ---------- -----FTVPE
Chtr_CRH62988.1                               ---------- ---------- ---------- ------SLST
PsAe_SQG59153.1                               AESGNSADEI TSIMPPSGSL IDPARTSVDL VLAGEVNVPS
Codi_WP_003852190.1                           NEVGNSADEV TSITPSSGNL IDPAHTKVSL ILAGRVEVPS
Chtr_CRH93390.1                               ---------- ---------- ---------- -----ITMPD
Chtr_CPS17231.1                               ---------- ---------- ---------- --------KD
Chtr_CQB89058.1                               ---------- ---------- ---------- ------LEPD

                                              841
StAu_newman_A6QGC0.1                          --TTESVDVP YTG---KNDK SQKVKVYIKD -KDNDGSTEK
Stau_aureus_CAA73980.1                        --TTESVDVP YTG---KNDK SQKVKVYIKD -KDNDGSTEK
Ecoli_MQS28384                                --TTESVDVP YTG---KNDK SQKVKVYIKD -KDNDGSTEK
PsAe_MUK59325.1                               --TTESVDVP YTG---KNDK SQKVKVYIKD -KDNDGSTEK
Stau_SCT90678.1                               ---------- -TH---QMSK RQLNR----- ----------
Stpn_Bacilli_multispecies_WP_048762376.1      --YTETYHIP YTG---NDGE SQKVKIYIRD -KNNSGTQVS
PsAe_WP_150019979.1                           ---------- ---------- ---------- ----------
PsAe_WP_121410777.1                           ---------- ---------- ---------- ----------
Enfu_VFA68308.1                               --SSTGTTT- ---------- ---------- -SDSVSSSTD
Stpn_WP_160544498.1                           --SSSEEI-- ---------- ---------- -DTDSSNQTS
Stpn_Streptococcus_multispecies_WP_049527645.1 --SSSSTT-- ---------- ---------- ---HSSSTSS
PsAe_RUC36476.1                               ---------- ---------- ---------- ----------
Enfu_WP_016624302.1                           --ISGYTK-- ---------- ---------- -SEAQKYLSE
Limo_WP_003733096.1                           --VTKTFNIP YTPSDEENPQ PQKIQIYIQD -KDHSMTSAY
Listeria_multispecies_WP_003767439.1          --VTKTFNIP YTPSDPENPQ PQKVQIYIQD -KDHSMTSAY
Stpn_CJR66226.1                               --VREKVKIP YEP--ENEGD ELQVQIAVDD -ADHSISDTY
Stpn_COC44426.1                               --VKEKISIP YEP--EHEGE ELEVQIAIDD -KDHSISDTY
Stpn_CVM85423.1                               --VTEKIAIP YEP--ETEGQ EMNVQISIDD -AEHSISDLY
Stpn_CVM86034.1                               -PTPSASASP SPP-PTESPE P--------- -SESAEPSPS
Chtr_CQB85900.1                               LAVGTVTF-- ---------- ---------- -KQAKQVLEA
Chtr_CRH87370.1                               --NPDKEK-- ---------- ---------- ----------
Chtr_CRH89418.1                               -GLVGYDQ-- ---------- ---------- -AYVVQTLQE
PsAe_WP_121124925.1                           ---------- ---------- ---------- ----------
Errh_WP_016357241.1                           ---------- ---------- ---------- YSESSRPKEE
Errh_WP_173446533.1                           ---------- ---------- ---------- ----------
Chtr_CRH67572.1                               --ITGKQE-- ---------- ---------- -AEAKRILEA
Chtr_CRH88717.1                               --VVGKQR-- ---------- ---------- -DEATRILEE
PsAe_SQG56831.1                               --ITRLTV-- ---------- ---------- -ADAVRALNA
Codi_WP_003849886.1                           --ITRQTI-- ---------- ---------- -GDAVRILHD
Chtr_CRH62988.1                               --LVSKGM-- ---------- ---------D ATEASQKLSD
PsAe_SQG59153.1                               --VVGKKY-- ---------- ---------- -SEAKKILEE
Codi_WP_003852190.1                           --VVGKRY-- ---------- ---------- -AEAKRELEA
Chtr_CRH93390.1                               FGYMKVSY-- ---------- ---------- -AEARRQLQA
Chtr_CPS17231.1                               ---------- ---------- ---------- -DDHSLNNIY
Chtr_CQB89058.1                               ---------- ---------- ---------- -DDESWM---
```

```
                                            881
StAu_newman_A6QGC0.1                        GSFDITSDQR I--------- ---------- --DIPLRIEK
Stau_aureus_CAA73980.1                      GSFDITSDQR I--------- ---------- --DIPLRIEK
Ecoli_MQS28384                              GSFDITSDQR I--------- ---------- --DIPLRIEK
PsAe_MUK59325.1                             GSFDITSDQR I--------- ---------- --DIPLRIEK
Stau_SCT90678.1                             ---------- ---------- ---------- ----------
Stpn_Bacilli_multispecies_WP_048762376.1    QTYNIHKDKI I--------- ---------- --TIPLKIEQ
PsAe_WP_150019979.1                         ---------- ---------- ---------- --------AE
PsAe_WP_121410777.1                         ---------- ---------- ---------- ----------
Enfu_VFA68308.1                             ASTSDSSSTS T--------- ---------- --SSSTLPSD
Stpn_WP_160544498.1                         SSRSSSSSNH S--------- ---------- --TEATGDAN
Stpn_Streptococcus_multispecies_WP_049527645.1 STESTSSSTE T--------- ---------- --PTEATHAD
PsAe_RUC36476.1                             ---------- ---------- ---------- ----------
Enfu_WP_016624302.1                         IGADYMGHES Y----EFSDS VEKDKVIRTN -PAAGTDISK
Limo_WP_003733096.1                         REMSITQNTS V--------- ---------- --EITFQIEE
Listeria_multispecies_WP_003767439.1        REMNITQNTS V--------- ---------- --EVTFQIEE
Stpn_CJR66226.1                             EEFKIKEPTE R--------- ---------- --TIELKIEP
Stpn_COC44426.1                             DSFKIKEPTE K--------- ---------- --TIELKIDQ
Stpn_CVM85423.1                             ETFKITAPTE R--------- ---------- --TIKFKIEP
Stpn_CVM86034.1                             PSASATPSPE PT--TTETPS PTQSPSPTSA APSEPTGQPP
Chtr_CQB85900.1                             KGFKVVASDA S--------- AKDDDIVTGM SEKEGAKIDK
Chtr_CRH87370.1                             ---------- ---------- ---------- -EKEKEKEKE
Chtr_CRH89418.1                             LGLRTSVAEQ Y------SNS LEAGGVLALN -PTEGSVVAQ
PsAe_WP_121124925.1                         ---------- ---------- ---------- --------EK
Errh_WP_016357241.1                         ADEK------ ---------- --------EE KPSEPEEKPD
Errh_WP_173446533.1                         ---------- ---------- ---------- ----------
Chtr_CRH67572.1                             AGLNVTVQRV LGG-VFGTAR YTD------- -PDAGTVVKK
Chtr_CRH88717.1                             AGFNVAVKEL LGG-YFGTVR LQDT------ --AGGTKAKK
PsAe_SQG56831.1                             AGWTGTASKL TQGERVPTVS LSDQNLIASQ VPTAGTPLRK
Codi_WP_003849886.1                         AGWKGNASRL IQAAKVPTVA VTDQNLIASQ LPTPGTALRK
Chtr_CRH62988.1                             AGFGVRVKRQ P------DAL VKKDTVI--- --SFPDKAKK
PsAe_SQG59153.1                             AGFTVKGTGA K----KPSSR VYWQ------ -SPTGGRETP
Codi_WP_003852190.1                         AGFSVTTSGT S----KSSAR VYWQ------ -TPAGGRAEP
Chtr_CRH93390.1                             LGVSVSAIEK Q---TDSTVT ASTSDMVISQ YPSAGSVIDG
Chtr_CPS17231.1                             RDLYIKHDMS F--------- ---------- --SIPFNIRE
Chtr_CQB89058.1                             ---------- ---------- ---------- ----------

                                            921
StAu_newman_A6QGC0.1                        GKTASYIVKV DGKT------ -----VAEKE VSYDD-----
Stau_aureus_CAA73980.1                      GKTASYIVKV DGKT------ -----VAEKE VSYDD-----
Ecoli_MQS28384                              GKTASYIVKV DGKT------ -----VAEKE VSYDD-----
PsAe_MUK59325.1                             GKTASYIVK- ---------- ---------- ----------
Stau_SCT90678.1                             ---------- ---------- ---------- ----------
Stpn_Bacilli_multispecies_WP_048762376.1    GDSAGYTIEV DDNV------ -----IADKD IDYE------
PsAe_WP_150019979.1                         GQSS------ ---------- ---------- ----------
PsAe_WP_121410777.1                         ---------- ---------- ---------- ----------
Enfu_VFA68308.1                             STTNT----- ---------- --------GT ANNPLTQ---
Stpn_WP_160544498.1                         SQ-------- ---------- ---------- ---PETTD--
Stpn_Streptococcus_multispecies_WP_049527645.1 QQ-------- ---------- ---------- ----------
PsAe_RUC36476.1                             ---------- ---------- ---------- ----------
Enfu_WP_016624302.1                         GDVVSVVYSK GVDPAKE--S SSEEDSSSSS SEEKSSDTTT
Limo_WP_003733096.1                         GSSAGYKIIS DDKV------ -----IDEGT VPYPN-----
Listeria_multispecies_WP_003767439.1        GSSAGYKIIS DDKV------ -----IDEGT VPYPN-----
Stpn_CJR66226.1                             GQKGYYQVMV NNKV------ -----VSYKT IEYPKDE---
Stpn_COC44426.1                             GQKGYYQVMV DHKV------ -----VSYKT IEYPKDND--
Stpn_CVM85423.1                             GQKGYYQVTV DDKV------ -----VSSKT IEYPDDE---
Stpn_CVM86034.1                             STSPTATPVD PLPTLTP--- -----DPEPS IDPPPST---
Chtr_CQB85900.1                             GSTITLTVKS ATPPSDLTKP GTGLDLNKPS TEDPSTSTTT
Chtr_CRH87370.1                             GSGTT----- ---------- --------PP GETPSHN---
Chtr_CRH89418.1                             GSTVNLTVSK G--------- ------PQPA PPNPNNSG-Q
PsAe_WP_121124925.1                         KLTVTLEGTE ---------- ---------- ----------
Errh_WP_016357241.1                         GSN------- ---------- ---------- ----------
Errh_WP_173446533.1                         ---------- ---------- ---------- ----------
Chtr_CRH67572.1                             GSTVTLFIV- ---------- ---------- ----------
Chtr_CRH88717.1                             GSTITLTIV- ---------- ---------- ----------
PsAe_SQG56831.1                             DAPIEIHLYE ---------- -----FSLSA LTNPR-----
Codi_WP_003849886.1                         DAPIEIRLYE ---------- -----FNLAA LVPPAQH---
Chtr_CRH62988.1                             GQTVTLVVSD GLPLINDSH- ---------- ----------
PsAe_SQG59153.1                             GAEIKLRTIG S--------- ---------- ----------
Codi_WP_003852190.1                         GATIKLKTLS S--------- ---------- ----------
Chtr_CRH93390.1                             TVTLYVSVAS ---------- -----TSGTT STTGSSSTTK
Chtr_CPS17231.1                             GSGQLKVVRN GDTV------ -----LNEKV TK--------
Chtr_CQB89058.1                             ---------- ---------- ---------- ----------
```

```
                                                961
StAu_newman_A6QGC0.1                              ---------- -----V
Stau_aureus_CAA73980.1                            ---------- -----V
Ecoli_MQS28384                                    ---------- -----I
PsAe_MUK59325.1                                   ---------- ------
Stau_SCT90678.1                                   ---------- ------
Stpn_Bacilli_multispecies_WP_048762376.1          ---------- ------
PsAe_WP_150019979.1                               ---------- ------
PsAe_WP_121410777.1                               ---------- ------
Enfu_VFA68308.1                                   ---------- ------
Stpn_WP_160544498.1                               ---------- ------
Stpn_Streptococcus_multispecies_WP_049527645.1    ---------- ------
PsAe_RUC36476.1                                   ---------- ------
Enfu_WP_016624302.1                               TSDKAESTTA ESENKD
Limo_WP_003733096.1                               ---------- ------
Listeria_multispecies_WP_003767439.1              ---------- ------
Stpn_CJR66226.1                                   ---------- ------
Stpn_COC44426.1                                   ---------- ------
Stpn_CVM85423.1                                   ---------- ------
Stpn_CVM86034.1                                   ---------- ------
Chtr_CQB85900.1                                   TNTGL----- ------
Chtr_CRH87370.1                                   ---------- ------
Chtr_CRH89418.1                                   ANSGS----- ------
PsAe_WP_121124925.1                               ---------- ------
Errh_WP_016357241.1                               --------DR EA----
Errh_WP_173446533.1                               ---------- ------
Chtr_CRH67572.1                                   ---------- ------
Chtr_CRH88717.1                                   ---------- ------
PsAe_SQG56831.1                                   ---------- ------
Codi_WP_003849886.1                               ---------- ------
Chtr_CRH62988.1                                   ---------- ------
PsAe_SQG59153.1                                   ---------- ------
Codi_WP_003852190.1                               ---------- ------
Chtr_CRH93390.1                                   TTTGS--TTS E-----
Chtr_CPS17231.1                                   ---------- ------
Chtr_CQB89058.1                                   ---------- ------
```
