# Supplementary material for: Synthesis and Antibacterial Evaluation of New Pyrazolo[3,4-d]pyrimidines Kinase Inhibitors
Source: Molecules. 2020 Nov 16;25(22):5354. doi: 10.3390/molecules25225354 (PMC7696985; doi:10.3390/molecules25225354)
Supplement: Supplementary file 1 [file molecules-25-05354-s001.zip › Supplementari Materials.docx]

Supplementary Materials

Synthesis and Antibacterial Evaluation of New Pyrazolo[3,4-*d*]pyrimidines Kinase Inhibitors

Chiara Greco ^1,^ ^†^, Rosa Catania ^2, †^, Dario Leonardo Balacco ^3^, Vincenzo Taresco ^4^, Francesca Musumeci ^1^, Cameron Alexander ^5^, Alan Huett ^2,^* and Silvia Schenone ^1,^*

^1^ Dipartimento di Farmacia, Università di Genova, Viale Benedetto XV 3, 16132 Genova, Italy; [greco.phd@difar.unige.it](mailto:greco.phd@difar.unige.it)

^2^ School of Life Sciences, University of Nottingham, Nottingham NG7 2UH, United Kingdom; [rosa.catania1@nottingham.ac.uk](mailto:rosa.catania1@nottingham.ac.uk)

^3^ School of Dentistry, Institute of Clinical Sciences, College of Medical and Dental Sciences, University of Birmingham, Birmingham B5 7EG, United Kingdom; [d.l.balacco@bham.ac](mailto:d.l.balacco@bham.ac).uk

^4^ School of Chemistry, University of Nottingham, University Park, Nottingham NG7 2RD, UK; vincenzo.taresco@nottingham.ac.uk

^5^ School of Pharmacy, University of Nottingham, University Park, Nottingham NG7 2RD, U.K

***** Correspondence: [Alan.Huett@nottingham.ac.uk](mailto:Alan.Huett@nottingham.ac.uk) (A.H.); schenone@difar.unige.it (S.S.).

† These authors contributed equally to this work.

**Table S1** The table reports the species name and their taxonomic identifier for the 10 organisms analysed.

| **Species** | **Taxonomy ID** |
| --- | --- |
| *Staphylococcus aureus st.* Newman | 426430 |
| *Staphylococcus aureus* | 1280 |
| *Escherichia coli* | 562 |
| *Pseudomonas aeruginosa* | 1126 |
| *Corynebacterium diphtheriae* | 1717 |
| *Enterococcus faecalis* | 1351 |
| *Erysipelothrix rhusiopathiae* | 1648 |
| *Listeria monocytogenes* | 1639 |
| *Streptococcus pneumoniae* | 1313 |
| *Chlamydia trachomatis* | 813 |

**Figure S1** Alignment of PASTA domain containing proteins – See separated file.

**Figure S2** Antibiotics doses screening for the selection of subinhibitory doses of Ampicillin (Amp) and Kanamycin (Kan) against S. aureus (top panels) and E. coli (low panels).
